# Light pollution and risk of diabetes: a systematic review and meta-analysis of observational studies

**DOI:** 10.3389/fpubh.2026.1709841

**Published:** 2026-02-13

**Authors:** Mengxiang Zhang, Yafei Xie, Dexuan Hu, Jianguo Xu, Junfei Wang, Lingdi Yang, Jinhui Tian, Bin Li

**Affiliations:** 1 The First School of Clinical Medicine, Lanzhou University, Lanzhou, China; 2 Department of Intensive Care Medicine, The First School of Clinical Medicine, Lanzhou University, Lanzhou, China; 3 Evidence-Based Medicine Center, School of Basic Medical Sciences, Lanzhou University, Lanzhou, China; 4 Department of Thoracic Surgery, West China Hospital, Sichuan University, Chengdu, China; 5 Chongqing Medical University, Chongqing, China; 6 Population Health Research Institute, Hamilton, ON, Canada; 7 Jingtai County Hospital of Traditional Chinese Medicine, Baiyin, China; 8 Key laboratory of Evidence-based Medicine of Gansu Province, Lanzhou University, Lanzhou, China

**Keywords:** diabetes, light pollution, meta-analysis, observational study, risk assessment, systematic review

## Abstract

**Background:**

Light pollution, characterized by excessive artificial light at night (LAN), is an emerging environmental risk factor with widespread impacts on human health. While its disruption of circadian rhythms is well-documented, its specific link to metabolic disorders like diabetes remains poorly synthesized.

**Objective:**

To systematically evaluate and quantify the association between light pollution exposure (both indoor and outdoor) and the risk of diabetes mellitus based on existing observational studies.

**Methods:**

We searched PubMed, Web of Science, Scopus, Embase, and CINAHL on January 9, 2024, and manually supplemented with citation searches. Two researchers independently screened literature and extracted data. Study quality was evaluated using the AHRQ and NOS scales. Random-effects meta-analyses synthesized risk estimates, with heterogeneity measured by I^2^. Publication bias was assessed using funnel plots and Beeg's test. Subgroup analyses were conducted based on the severity and type of light pollution. The GRADE method assessed evidence credibility.

**Results:**

Out of 2,115 identified studies, six were included in the quantitative synthesis. Light pollution exposure was associated with a 31% increase in diabetes risk (OR: 1.31, 95% CI: 1.13–1.33; GRADE: moderate). Subgroup analyses showed significant correlations with severe light pollution (OR: 1.19, 95% CI: 1.14–1.24; GRADE: moderate), low to moderate light pollution (OR: 1.10, 95% CI: 1.06–1.14; GRADE: moderate), and indoor light pollution (OR: 1.66, 95% CI: 1.15–2.39; GRADE: moderate). Heterogeneity sources included sample size, light pollution type, and study quality.

**Conclusion:**

Exposure to light pollution is positively associated with increased diabetes risk, particularly with indoor light pollution. However, the limited number of included studies underscores the need for more prospective cohort studies with standardized exposure assessment and covariate adjustment.

**Systematic Review Registration:**

PROSPERO https://www.crd.york.ac.uk/prospero/, identifier CRD42024551969.

## Highlights

- Provides the best available evidence on the association between light pollution and diabetes from various perspectives.
- Classify light pollution as artificial daylight, neon lights, reflected sunlight and occupational UV/IR radiation.
- Covers exposure assessment, data analysis methods, and covariate adjustment.
- Covers exposure assessment, data analysis methods, and covariate adjustment.

## Introduction

With the advent of industrial civilization, people are increasingly exposed to light (1). In recent years, the issue of light pollution has gained recognition due to an increase in the number of studies on the risks associated with light pollution and health (2). In accordance with the definition of light pollution as defined by the International Dark-Sky Association (2022; the inappropriate or excessive use of artificial light that can have serious environmental consequences for humans, wildlife, and our climate) (3), It includes four types: artificial daylight, color light pollution (neon lights), white bright pollution (reflected sunlight), and occupational UV and infrared radiation (2). It can be estimated that approximately 80% of the global population is exposed to various forms of light pollution, with the most notable impact being the alteration of natural night-time lighting levels caused by artificial light sources (4). There is growing evidence that light pollution may have a significant impact on a wide range of diseases, including obesity (5), cancer (6, 7), mental disorders (8), sleep disorders (9), cardiometabolic disease (10), and diabetes (11–16).

Diabetes, a chronic disease, affects over 11.1% of the global population, projected to reach 13% in 25 years (17). Animal studies show that light pollution induces glucose intolerance in mice (18, 19). However, some studies suggest it only alters circadian rhythms without affecting glucose tolerance (20), especially among night shift workers (21). Cross-sectional studies indicate a significant association between light pollution and diabetes (11, 12, 16), though some disagree (22–24). Prospective studies suggest long-term light pollution exposure increases diabetes risk (13–15).

To the best of our knowledge, only one review (2) and one meta-analysis (25) have been published on the relationship between light pollution and diabetes. Our findings align with previous studies; however, the prior review lacked a meta-analysis, and the earlier meta-analysis included fewer studies and did not conduct subgroup or sensitivity analyses. Therefore, we have performed a more comprehensive meta-analysis to provide the most robust epidemiological evidence on the association between light pollution and diabetes. Our aim is to offer policymakers, healthcare professionals, and patients a solid foundation for decision-making in diabetes prevention.

## Methods

We conducted this review following PRISMA guidelines (26) and registered it with Prospective Register of Systematic Reviews (PROSPERO), CRD42024551969.

### Eligibility criteria

Based on the research questions, we identified key factors (Patient, Intervention, Comparison, Outcome, Study design, PICOS) for the review: *Participants*: the subjects in the cross-sectional study were adults, while those in the cohort study were adults with undiagnosed diabetes. Both studies excluded minors and animal studies. *Intervention (Exposure)*: no restrictions on the type of light pollution of interest. *Comparison*: different levels of exposure. *Outcomes*: outcomes of interest were determined by clinical diagnostic assessment from medical records, and quantitative effect estimates with 95% confidence interval (CI) are provided. *Study types*: we included observational studies and excluded case reports, non-original reports, ecological studies, conference abstracts, and reviews.

### Data source and data collection

We searched PubMed, Web of Science, Scopus, Embase, and Cumulative Index to Nursing and Allied Health Literature (CINAHL) on January 9, 2024, using terms like “light pollution”, “diabetes mellitus,” and “observational studies” without restrictions. Reference lists of relevant reviews were also checked. The detailed search strategy was presented in Appendix Table 1. Two independent researchers extracted data, with a third resolving disagreements. Data included general characteristics, explanation of light pollution, exposure assessment, explanation of health outcome, statistical approaches and associations between exposure to outcome. Unextractable data were marked as NA.

### Study selection process

The search results were imported into the Rayyan online literature screening platform (27). After duplicates were removed, two researchers independently screened titles and abstracts. After cross-checking, full-text reading by two independent researchers to screen the remaining articles. Disagreements were resolved by a third researcher.

### Quality assessment

We assessed risk of bias for cohort studies using the Newcastle-Ottawa scale (NOS) and for cross-sectional studies using the Agency for Healthcare Research and Quality scale (AHRQ) (28, 29). The NOS evaluates three domains: participant selection, comparability of exposure and control groups, and outcome determination. Studies are rated with up to nine stars: two stars for “comparability of groups on the basis of design or analysis” and one star for each of the remaining seven entries. Scores ≥7^*^ indicate high quality, 5–6^*^ indicate medium quality, and ≤ 4^*^ indicate low quality. The AHRQ scale assesses five domains: selection bias, implementation bias, measurement bias, follow-up bias, and reporting bias, with a total of eleven items. Each item is scored as “yes” (one point) or “no/unclear” (zero points). Scores of 8–11 are high quality, 5–7 are moderate quality, and 0–4 are low quality.

We adapted these scales for our studies (Appendix Tables 3A, D). Two researchers independently assessed each study's risk of bias, with disagreements resolved by a third researcher. The rationale for the ratings was documented (Appendix Tables 3C, F).

### Statistical analyses

A random-effects model synthesized risk estimates due to study heterogeneity (30). We extracted Odds Ratios (OR), Risk Ratios (RR), Hazard Ratios (HR), and Prevalence Ratios (PR) with 95% CIs from the most comprehensive models adjusted for confounders. Studies with clear diabetes definitions were included. Disparate effect sizes were converted to OR uniformly (13–16). We combined the effect sizes of the three studies as the total light pollution exposure group (14–16). For outcomes employing disparate exposure dose groupings (14–16), we initially combined the grouped data by considering the lowest exposure dose group as the control group. In particular, the risk estimate corresponding to the total light pollution exposure in comparison to the lowest light pollution exposure category was employed to generate the pooled effect size. The results of the pooled analyses are presented in a forest plot. The degree of heterogeneity between studies was quantified using the I-squared (I^2^) index. I^2^ values of less than 25% were considered to indicate low heterogeneity, values greater than 75% indicated high heterogeneity, and values between 25 and 75% indicated moderate heterogeneity (31). Analyses were performed using R 4.4.0.

### Subgroup and sensitivity analysis

Subgroup analyses were based on condition severity, light pollution type, geographic location, sample size, study quality, and study type. Sensitivity analyses used case-by-case exclusion. Publication bias was assessed with Beeg funnel plots.

### Quality assessment of the body of evidence

Grading System for Assessment, Development and Evaluation of Recommendations (GRADE) was used to assess evidence quality. GRADE is divided into five downgrades and three upgrades, and classifies evidence into four levels: high, moderate, low, and very low. We gave the review a preliminary rating of high, even though it included only observational studies, because it was not possible to blind to light pollution and therefore no case-control studies could be conducted (32). GRADE was adjusted for our study (Appendix Table 9A). Ratings were conducted by two researchers, with disagreements resolved by a third. The rationale for the ratings was documented (Appendix Table 9C).

## Results

### Studies selection and study characteristics

**Figure 1:**
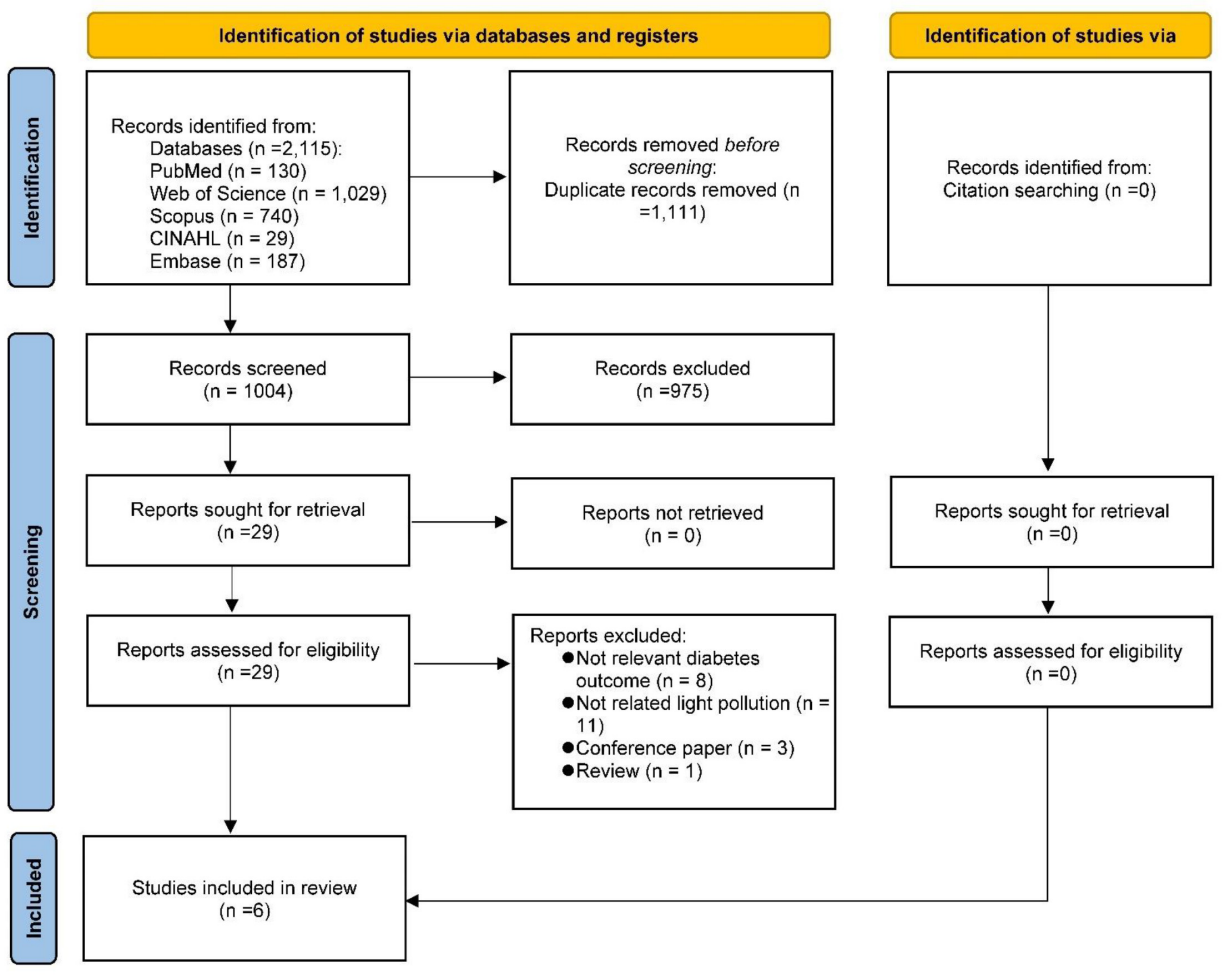
Flow diagram of study selection process.

The literature search and selection process are shown in Figure 1. From a total of 2,115 records, 1,004 remained after removing duplicates. Initial screening of titles and abstracts excluded 975 records that did not meet eligibility criteria. No additional relevant literature was found through citation searching. Of the 29 records reviewed in full, 23 were excluded based on eligibility criteria (Appendix Table 2). Six records were included in the quantitative assessment (11–16).

The brief characteristics of the included studies are illustrated in Table 1. A total of three cross-sectional studies (11, 12, 16) and three cohort studies (13–15) with a total of 645,010 participants were included. Study populations were from the USA (11), Japan (12, 13), UK (14, 15) and China (16). Three studies included only older adults over the age of 70 (11–13), two included middle-aged adults (14, 15), and one included all-age adults (16). Four studies examined indoor light pollution (11–14), one investigated blue light (14), one investigated blue light (15, 16). Three studies had large sample sizes (98,658 to 471,686 participants) (14–16), while three were small sample studies (11–13). Detailed characteristics are in Appendix Table 4.

**Table 1:** Brief general characteristics of the studies included in the review.

**Study ID (country)**	**participants (mean age, years)**	**Exposure (type of light pollution)**	**Outcome**	**Adjustment for covariates**	**Type of effect size**	**Effect size [95% CI]**	**NOS or AHRQ assessment**
Kim, M, 2023 (11) United States	552 (72, 5)	Indoor LAN	risk of diabetes	age, sex, race, season.	OR	2 [1.19–3.43]	AHRQ: 8 high quality
Obayashi, K, 2014 (12) Japan	513 (72.7 ± 6.5)	Indoor Elavg (evening light)	risk of diabetes	gender, BMI, duration in bed and Nlavg.	OR	1.13 [0.36–3.67]	AHRQ: 4 low quality
Obayashi, K, 2020 (13) Japan	678 (10.6 ± 6.6)	Indoor LAN	risk of diabetes	age, gender, current smoking status, alcohol consumption, education, household income, BMI, hypertension, caloric intake, daytime physical activity, bedtime, rise time, daytime light exposure, actigraphic TST, SE.	RR	3.17 [1.32–7.63]	NOS: 6^*^ moderate quality
Wang, C, 2023 (14) United Kingdom	471,686 (56.3 ± 8.11)	Indoor blue light	risk of diabetes	age, sex, ethnicity, education level, income level, Townsend index, smoking, alcohol use, healthy diet, body mass index, physical activity, hypertensive disorders, sleep quality score, time spent outdoors in the summer, time spent outdoors in the winter, cardiovascular disease, and cancer.	HR	1.20 [1.17–1.24]^a^	NOS: 7^*^ high quality
Xu, Z, 2023 (15) United Kingdom	283,374 (55.8 ± 8.10)	Outdoor LAN	risk of diabetes	age, sex, ethnicity, region, education, economic activity, household income, income score, housing score, shift work, smoking status, drink frequency, physical activity, sedentary time, health diet score, PM2.5, NO2, night noise, PRS, a higher score means higher genetic predisposition, population density in home geolocation with a buffer 1 km^2^.	HR	1.09 [1.03–1.15]^a^	NOS: 8^*^ high quality
Zheng, R, 2023 (16) China	98,658 (NA)	Outdoor LAN	risk of diabetes	age, sex, education, smoking status, drinking status, physical activity, family history of diabetes, household income, urban/rural living, taking antihypertensive medications, taking lipid-lowering medications, BMI.	PR	1.12 [1.00–1.24]^a^	AHRQ: 9 high quality

^*^Evening light (ELavg), the average light intensity during the 4 h prior to bedtime; daytime light (DLavg), the average light intensity during the out-of-bed period; TST, total sleep time; SE, sleep efficiency; PR, the prevalence ratio.

^a^Post-treatment effect size.

### Study quality and risk of bias assessment

Using the NOS risk of bias assessment, all studies scored ≥6^*^. One study scored 8^*^(15), one scored 7^*^(14) (both high-quality), and one study at 6^*^(13) (moderate-quality). For cross-sectional studies assessed with the AHRQ scale, two were high-quality [scores of 8 (11) and 9 (16), respectively] and one was a low-quality study [score of 4 (12)]. Detailed scores are in Appendix Tables 3B, E.

### Exposure explanation and assessment

Different studies had varied interpretations and metrics for light pollution exposure, consistent with our definition (Appendix Table 5). Criteria for determining exposure levels differed (Appendix Table 6). One study divided light pollution into yes and no, using the mean of the least active 5 h, calculated by averaging every minute over a 24-h period (11). One study considered an average nighttime light intensity of ≥5 lux as being exposed to light pollution (13). One study used a questionnaire to confirm the strength of blue light exposure (14). Three studies defined the intensity of light pollution exposure through different intervals by using quartiles or quintiles describing light intensity (12, 15, 16). We defined the 25%−75% interval of light pollution studied using quartiles (15) as moderate intensity light pollution and the 75%−100% interval as severe light pollution. We defined the 20–60 per cent interval of light pollution studied using quintiles (16) as moderate intensity light pollution and the 60–100 per cent interval as severe light pollution. Measurement methods included portable wrist recorders (11–13), questionnaires (14) and satellite data (15, 16) for outdoor light intensity.

### Statistical approaches

We summarized the statistical methods used for the data in all studies, as shown in Appendix Table 7. Multivariable model for three studies (11, 12, 16), Poisson regression models for one study (13), and Cox proportional hazards model or its modified version for two studies (14, 15). Three trials performed stratified analyses (11, 14, 15). All studies adjusted for covariates, included sex, age, BMI, smoking, region, income, and education, among others. Sensitivity analyses were performed in all but two studies (12, 16).

### Outcomes of the association between light pollution and diabetes

**Figure 2:**
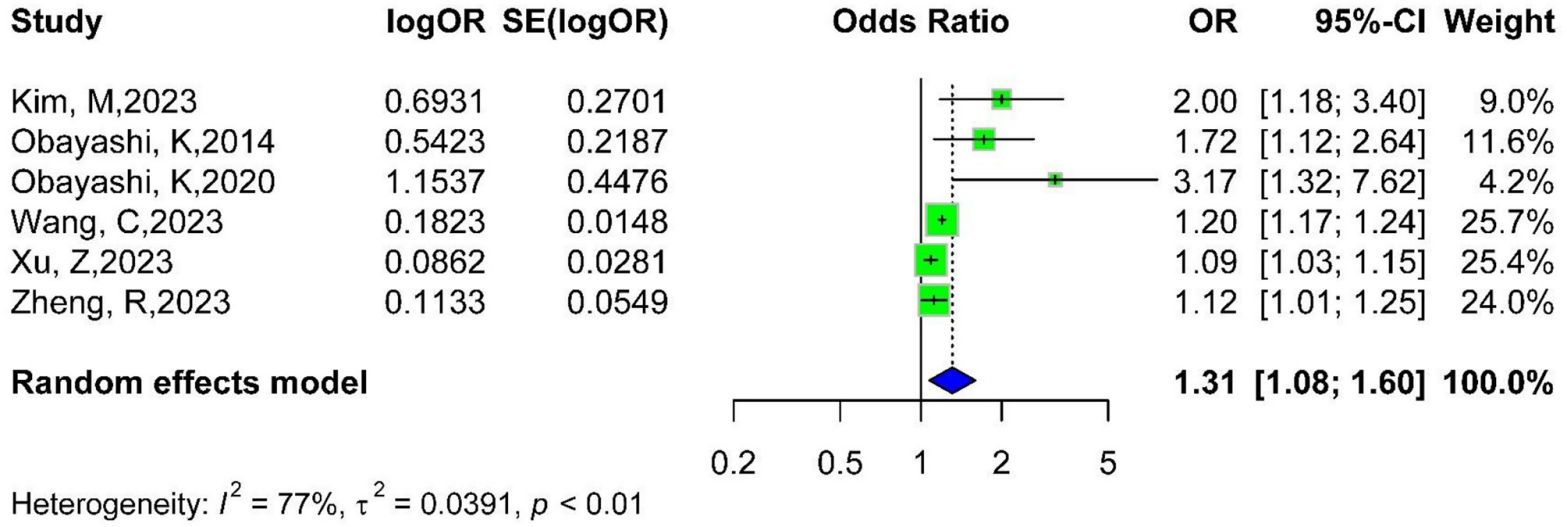
Forest plot of the overall relationship between light pollution and diabetes risk.

The relationship between light pollution exposure and diabetes outcomes is shown in Appendix Table 8. The prevalence of diabetes was reported in all studies. We combined the effect sizes of the total light pollution exposure group obtained indirectly from the pooling with the effect sizes of the total light pollution exposure group obtained directly from the original studies (Figure 2), and shown an OR of 1.31 (95% CI: 1.08–1.60), suggesting a 31% increased risk of diabetes. Heterogeneity was high (I^2^ = 77%).

#### Severity of light pollution

**Figure 3:**
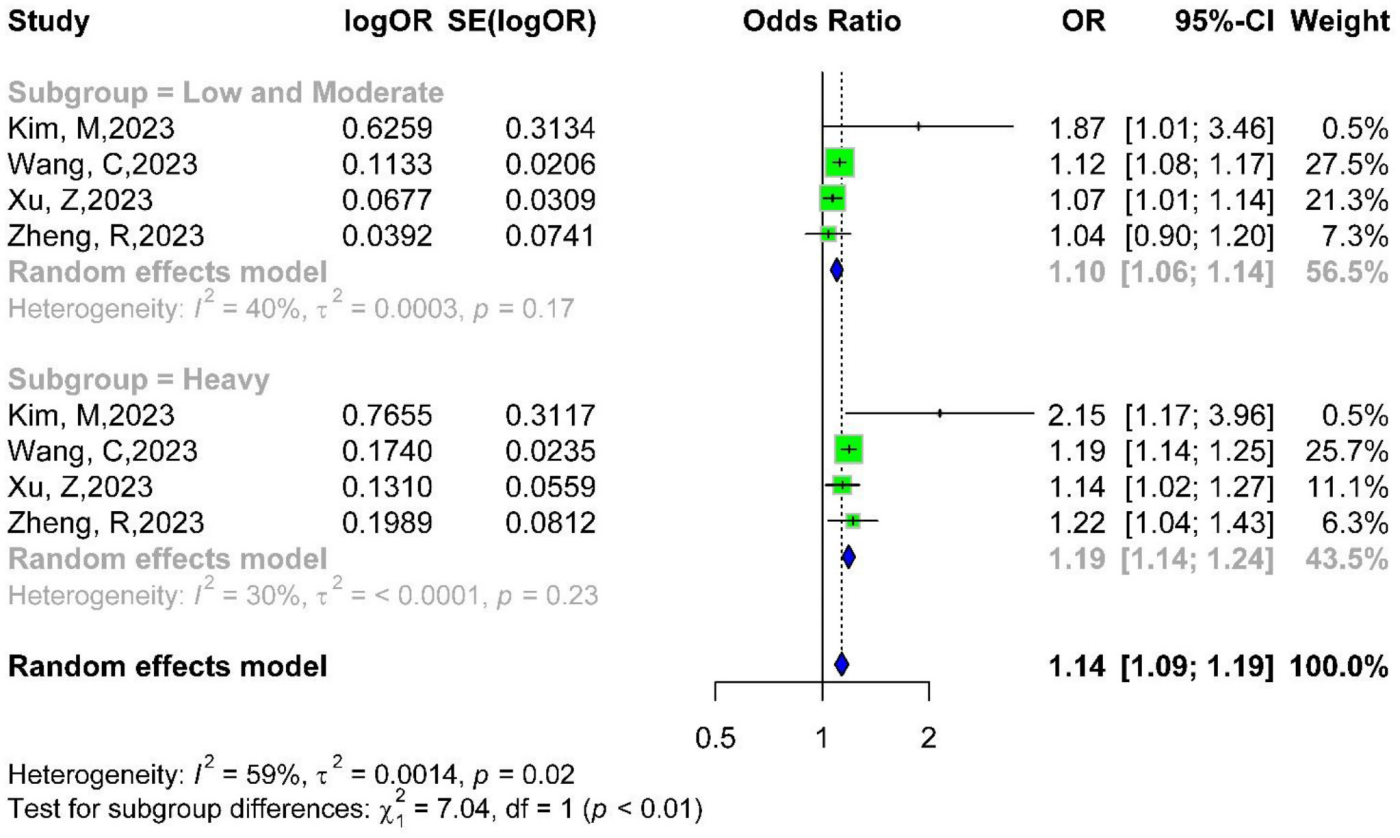
Subgroup forest plot of light pollution severity and diabetes risk.

Four studies were able to use light pollution exposure as a basis for subgroup analyses (11, 14–16). OR for low and moderate exposure was 1.10 (95% CI: 1.06–1.14), and for severe exposure, OR was 1.19 (95% CI: 1.14–1.24). There were significant intergroup differences (*P* < 0.01), indicating an increased risk with higher light pollution severity (Figure 3).

#### Type of light pollution

**Figure 4:**
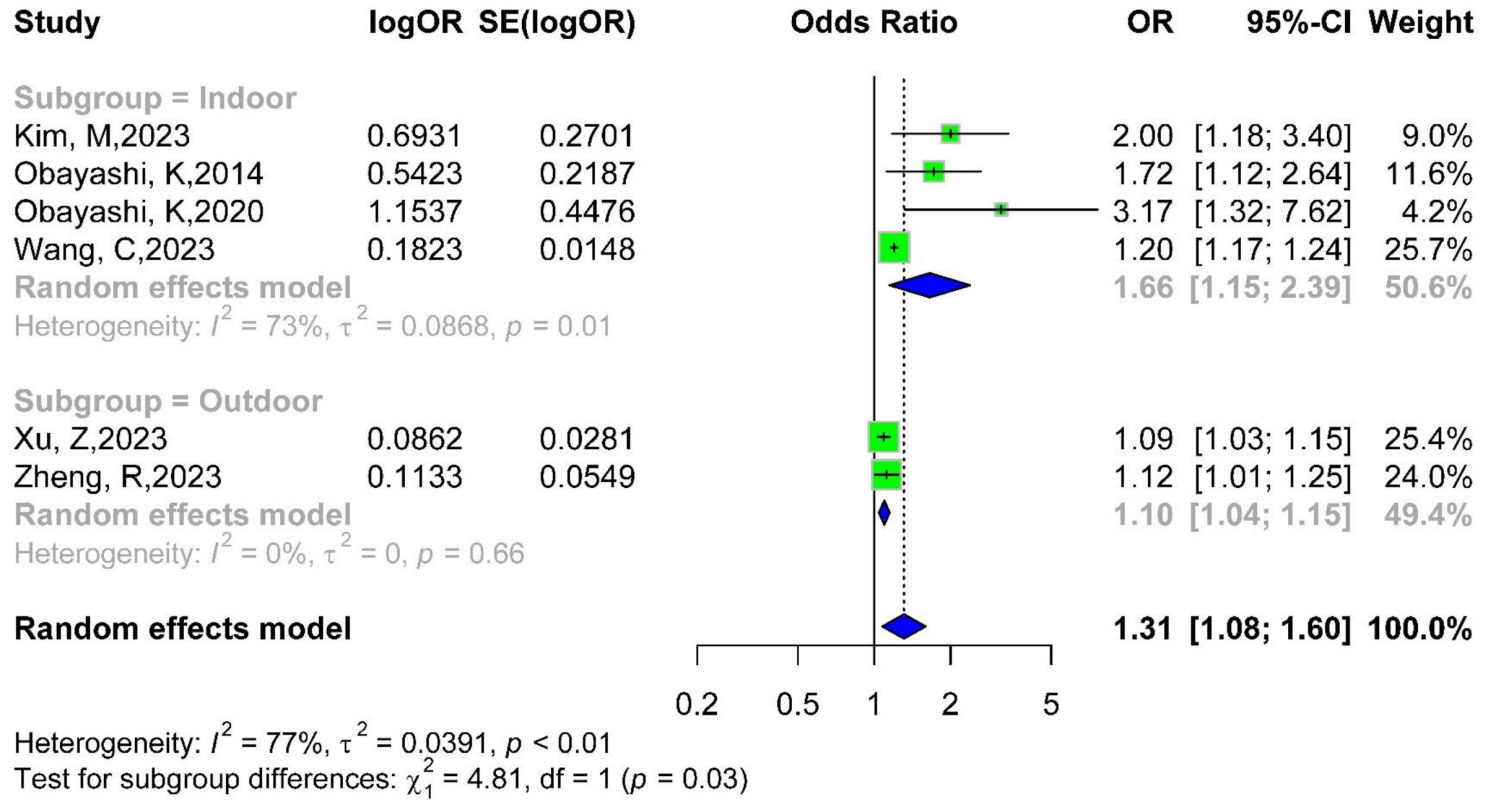
Subgroup forest plot of light pollution study type and diabetes risk.

Subgroup analysis by light pollution type showed an OR of 1.66 (95% CI: 1.15–2.39) for indoor light pollution, indicating a 66% increased risk (11–14). For outdoor light pollution, OR was 1.10 (95% CI: 1.04–1.15), indicating a 10% increased risk (15, 16). Intergroup differences were significant (*P* = 0.03), suggesting indoor light pollution is more likely to trigger diabetes (Figure 4).

#### Area

Geographic subgroup analysis indicated no significant differences (*P* = 0.25), suggesting geography is not a source of heterogeneity.

#### Study type

Subgroup analysis by study type also showed no significant differences (*P* = 0.24), indicating study type is not a source of heterogeneity.

#### Study quality

Subgroup analysis by study quality revealed significant differences (*P* = 0.04). High-quality studies showed an OR of 1.15 (95% CI: 1.08–1.23) (11, 14–16), while low- and medium-quality studies showed an OR of 1.96 (95% CI: 1.43–2.67) (12, 13), while low- and medium-quality studies showed an OR of 1.96 (95% CI: 1.43–2.67).

#### Sample size

Significant differences were found in subgroup analyses by sample size (*P* < 0.01). Small sample studies showed an OR of 1.96 (95% CI: 1.43–2.67) (11–13) and large sample studies showed an OR of 1.06 (95% CI: 1.03–1.09) (14–16), suggesting small-sample studies may increase the overall effect size.

#### Heterogeneity analysis

Subgroup analyses identified severity, type of light pollution, study quality, and sample size as potential sources of heterogeneity (Table 2 and Appendix Figure 1).

**Table 2:** Subgroup analysis of the pooled odds ratio between light pollution and diabetes.

**Moderators**	***N***	**Sample size**	**ORs (95%CI)**	***P* ^a^**	**Heterogeneity**		
					**I^2^ (%)**	***t* ^2^**	***P* ^b^**
**Severity**							
Low and moderate	4	452,999	1.10 [1.06–1.14]	<0.01	40%	0.0003	0.17
Heavy	4	190,948	1.19 [1.14–1.24]		30%	<0.0001	0.23
**Area**							
Europe and America	3	569,440	1.17 [1.05–1.31]	0.25	85%	0.0062	<0.01
Asia	3	75,570	1.60 [0.95–2.70]		77%	0.1553	0.01
**Light pollution type**							
Indoor	4	357,973	1.66 [1.15–2.39]	0.03	73%	0.0868	0.01
Outdoor	2	287,037	1.10 [1.04–1.15]		0%	0	0.66
**Study type**							
Cohort study	3	569,693	1.16 [1.05–1.28]	0.24	86%	0.0048	<0.01
Cross-sectional study	3	75,317	1.46 [1.01–2.13]		74%	0.076	0.02
**Sample quantity**							
Small sample quantity	3	1,360	1.96 [1.43–2.67]	<0.01	0%	0	0.47
Large sample quantity	3	643,650	1.14 [1.07–1.22]		80%	0.0025	<0.01
**Quality**							
High	4	643,947	1.15 [1.08–1.23]	0.04	78%	0.0026	<0.01
Low and moderate	2	1,063	2.06 [1.19–3.56]		34%	0.0628	0.22

^a^P-value for the between-subgroup difference.

^b^P-value for the heterogeneity within subgroups by Q-test.

We believe that the heterogeneity may be caused: a) clinical heterogeneity: differences in the age of the included populations, types of light pollution exposure, differences in light pollution controls, and types of studies; and b) methodological heterogeneity: differences in sample sizes, differences in the regions from which the samples were drawn, and differences in the quality of the studies.

#### Sensitivity analysis

Cull-by-cull sensitivity analyses indicated moderate to high heterogeneity (I^2^ values: 68%−81%). Results were robust, but some studies had greater influence on overall effect size and heterogeneity (Appendix Figure 2).

### Publication bias and GRADE assessment

**Figure 5:**
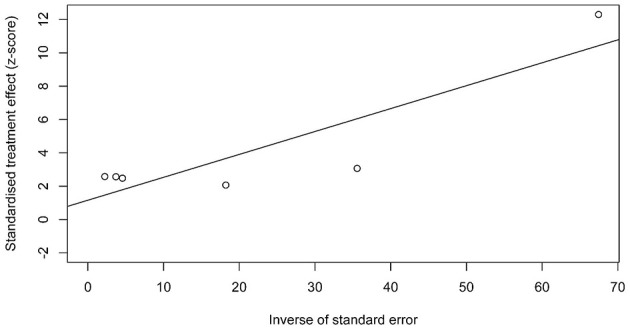
Funnel plot showing the risk of publication bias in the meta-analysis.

This was done even though publication bias testing was not required for the inclusion of less than 10 papers (33). Funnel plot analysis for publication bias showed no significant evidence of skewness (*t* = 0.99, *P* = 0.3783; Figure 5). GRADE assessment rated evidence as medium quality for most outcomes, high quality for outdoor light pollution, and low quality for Asia-related diabetes incidence (Appendix Table 9B).

## Discussion

This meta-analysis synthesizes the impact of light pollution on diabetes risk, incorporating six studies (three cohort and three cross-sectional). Four of these studies were published in 2023 (11, 14–16), reflecting growing interest in this area. Despite variations in light pollution exposure, the overall risk of developing diabetes was 31% higher among those exposed to light pollution. Indoor light pollution exposure resulted in a 66% increased risk, compared to a 10% increase with outdoor light pollution, indicating that indoor exposure poses a more significant risk.

Our findings are consistent with previous speculations. To date, there is literature on the effects of light pollution and metabolic syndrome, which has found that outdoor light pollution exposure is associated with metabolic syndrome (20). Furthermore, epidemiological data shows that night shift work increases the prevalence of diabetes (21, 34). We hypothesize that light pollution is also associated with diabetes. In recent cross-sectional studies, three studies concluded that light pollution was associated with an increased risk of diabetes (11, 12, 16), while three concluded that light pollution was not associated with diagnostic markers of diabetes (22–24). The latter were excluded from the analysis because they only collected data on relevant markers [plasma glucose, HbA1c (glycated hemoglobin) and so on] and did not have definitive diabetes diagnostic data. It is hypothesized that the main reason for this inconsistency is that more clinical information is needed to confirm a diagnosis of diabetes. Furthermore, three cohort studies have indicated that light pollution may be associated with an increased incidence of diabetes (13–15).

An interesting finding was that indoor light pollution had a considerably greater impact on diabetes risk than outdoor light pollution. Specifically, exposure to indoor light pollution was associated with a 66% increased risk of diabetes, while exposure to outdoor light pollution was associated with a 10% increased risk of diabetes. This is consistent with the findings obtained in previous meta-analyses (10). One possible explanation for this difference is that several studies included in the analysis were conducted among older adults, who generally spend more time indoors and are therefore more susceptible to indoor light exposure. In this review, exposure was measured in three ways: a) wrist-mounted photometric devices; b) use of questionnaires to determine the intensity of exposure; and c) use of satellite data with matching to specific individuals. However, data from questionnaires and satellites may not reflect the intensity of light pollution as accurately as wrist photometric devices (9). Consequently, the effect of light pollution on the prevalence of diabetes may be underestimated if the type of light pollution is not taken into account. Heterogeneity among studies might stem from different definitions of light pollution exposure and control groups. For example, Studies used varying criteria to define “no light pollution,” including illuminance < 5 lux (13), the lowest quartile or quintile of light exposure (15, 16), or exposure durations under1 h (14). Different studies employ either duration of illumination or light intensity to define light pollution. These varying definitions contribute to heterogeneity.

It is worth noting that the sample sizes of almost all studies that used wrist-mounted photometric devices to collect data were small, probably for a number of reasons, including cost. Subgroup analyses indicated that sample size, type of light pollution, and study quality were significant sources of heterogeneity. Additionally, although subgroup analyses did not reveal differences in the type of study, inherent limitations in cross-sectional studies may have contributed to the observed heterogeneity (35). It is postulated that this may be due to the fact that fewer literatures are included, resulting in no differences in the results of the subgroup analyses.

It is important to be aware of the limitations of this study. Firstly, the number of studies included in the literature (*n* = 6) was relatively limited. However, meta-analysis was considered acceptable and valid when *n* > 4 studies were included in the study (36). Second, while no significant publication bias was detected, it may still exist due to the limited number of studies. Third, the lack of detailed data prevented subgroup analysis by factors like population, age, and socio-economic status. Fourth, differences in how studies controlled for confounders could affect the results. Despite these limitations, the strengths of this study are worth mentioning. This study offers a comprehensive analysis of the latest data in the field, employing standardized criteria for literature search, study selection, quality assessment, data extraction, evidence synthesis, and credibility assessment. This approach enhances the study's transparency, rigor, and reliability. Additionally, we conducted in-depth subgroup and sensitivity analyses, thoroughly exploring sources of heterogeneity. Consequently, the significant positive association between light pollution and diabetes risk presented here is robust and reliable.

Although there is significant evidence linking light pollution to diabetes, the variation in study designs and methodologies creates challenges for data integration. Future research should adopt standardized methods for exposure assessment. We could not perform a dose-response meta-analysis due to insufficient data, and more high-quality prospective studies are needed to confirm these findings.

## Conclusion

This systematic review and meta-analysis suggest a potential positive association between light pollution exposure and the risk of diabetes, with higher risks observed under more severe light pollution, particularly in indoor settings. However, this conclusion should be interpreted with caution due to the limited number of studies included, the heterogeneity of study populations, and the inconsistency in exposure assessment methods. Further large-scale, standardized longitudinal studies with objective measurements of light exposure are warranted to clarify the causal relationship and underlying mechanisms.

## Data Availability

The original contributions presented in the study are included in the article/Supplementary material, further inquiries can be directed to the corresponding authors.
