# Supplementary material for: Light pollution and risk of diabetes: a systematic review and meta-analysis of observational studies
Source: Front Public Health. 2026 Feb 13;14:1709841. doi: 10.3389/fpubh.2026.1709841 (PMC12946003; doi:10.3389/fpubh.2026.1709841)

# Supplement appendix

Content

**Appendix Table 1: Search strategy**

**Appendix Table 2****: Excluded studies and reasons for their exclusion after full texts assessment with additional reference**

**Appendix Table 3: Quality assessment of the included studies**

**Appendix Table 4: General characteristics for the studies included in the review**

**Appendix Table 5: Explanation of light pollution in the studies included in the review**

Appendix Table 6: **Exposure assessment in the studies included in the review**

**Appendix Table 7: Statistical approaches in the studies included in the review**

**Appendix Table 8: Associations between exposure to light pollution and diabetes outcome**

**Appendix Table 9: GRADE assessment for the association between light pollution and diabetes outcome Appendix figure 1: Light pollution and diabetes incidence subgroup forest plots**

Appendix Table 10: List of abbreviations

**Appendix figure 2: Publication bias and funnel plots**

**Appendix figure 3: Sensitivity analysis forest plot**

Appendix Table 1: Search strategy

**Table 1A. PubMed search**

| Search | Query | Results | Time |
| --- | --- | --- | --- |
| #18 | Search: #3 AND #6 AND #17 | 130 | 07:47:27 |
| #17 | Search: #9 OR #12 OR #15 OR #16 | 5,188,764 | 07:46:23 |
| #16 | Search: Observational[Title/Abstract] | 280,000 | 07:43:06 |
| #15 | Search: #13 OR #14 | 1,355,565 | 07:42:25 |
| #14 | Search: ((cross-sectional[Title/Abstract]) OR (prevalence[Title/Abstract])) OR (transversal[Title/Abstract]) | 1,249,136 | 07:42:19 |
| #13 | Search: Cross-Sectional Studies[MeSH Terms] | 488,037 | 07:41:39 |
| #12 | Search: #10 OR #11 | 1,926,543 | 07:41:33 |
| #11 | Search: Case*[Title/Abstract] AND control*[Title/Abstract] | 627,271 | 07:40:52 |
| #10 | Search: case-control studies[MeSH Terms] | 1,470,642 | 07:40:26 |
| #9 | Search: #7 OR #8 | 3,477,147 | 07:39:16 |
| #8 | Search: Cohort[Title/Abstract] | 906,662 | 07:38:09 |
| #7 | Search: (((((cohort studies[MeSH Terms]) OR (longitudinal study[MeSH Terms])) OR (prospective study[MeSH Terms])) OR (follow up study[MeSH Terms])) OR (controlled clinical trial[Publication Type]) | 3,086,509 | 07:37:20 |
| #6 | Search: #4 OR #5 | 981,506 | 07:33:31 |
| #5 | Search: ((((((((((diabet*[Title/Abstract] )OR (DM1[Title/Abstract])) OR (T1DM[Title/Abstract])) OR (T2DM[Title/Abstract])) OR (T2D[Title/Abstract])) OR (T1D[Title/Abstract])) OR (T2D[Title/Abstract])) OR (MODY[Title/Abstract])) OR (impaired glucose tolerance[Title/Abstract])) OR (glucose intolerance[Title/Abstract])) OR (insulin* depend* [Title/Abstract])) OR (IDDM[Title/Abstract])) OR (non insulin* depend*[Title/Abstract])) OR (NIDDM[Title/Abstract])) OR (noninsulin dependent[Title/Abstract])) OR (non insulindependent[Title/Abstract])) OR (Noninsulindependent[Title/Abstract])) OR (Insulin Resistance[Title/Abstract])) OR (Glucosuria[Title/Abstract])) OR (insulin sensitivity[Title/Abstract])) | 901,149 | 07:27:43 |
| #4 | Search: diabetes mellitus[MeSH Terms] OR Insulin Resistance[MeSH Terms] | 599,110 | 07:26:56 |
| #3 | Search: #1 OR #2 | 44381 | 07:25:36 |
| #2 | Search: Light Pollution[Title/Abstract] OR Artificial Light at Night[Title/Abstract] OR Light at Night[Title/Abstract] OR Night Light[Title/Abstract] OR Nighttime Light[Title/Abstract] OR Environmental Light*[Title/Abstract] OR Domestic Light[Title/Abstract] OR Artificial Night Lighting[Title/Abstract] OR Skyglow[Title/Abstract] OR Artificial Night Sky Brightness[Title/Abstract] OR Light Trespass[Title/Abstract] OR Blue Light*[Title/Abstract] OR Light Emitting Diode*[Title/Abstract] OR Light Exposure[Title/Abstract] OR Glare*[Title/Abstract] | 44,377 | 07:23:45 |
| #1 | Search: Light Pollution[MeSH Terms] | 141 | 07:22:21 |

**Table 1B. Web of Science search**

| Search | Query | Results | Date |
| --- | --- | --- | --- |
| #8 | #1 AND #2 AND #7 and Preprint Citation Index (Exclude – Database) | 1029 | 08-jan-24 |
| #7 | #3 OR #4 OR #5 OR #6 and Preprint Citation Index (Exclude – Database) | 8371235 | 08-jan-24 |
| #6 | TS=(Observational) and Preprint Citation Index (Exclude – Database) | 461886 | 08-jan-24 |
| #5 | ((TS=(cross-sectional)) OR TS=(prevalence)) OR TS=(transversal) and Preprint Citation Index (Exclude – Database) | 2375068 | 08-jan-24 |
| #4 | TS=(Case* and control*) and Preprint Citation Index (Exclude – Database) | 1755257 | 08-jan-24 |
| #3 | (((TS=(longitudinal)) OR TS=(prospective)) OR TS=(follow up)) OR TS=(Cohort*) and Preprint Citation Index (Exclude – Database) | 4975096 | 08-jan-24 |
| #2 | ((((((((((((((((((TS=(diabet*)) OR TS=(DM)) OR TS=(T1DM )) OR TS=(T2DM )) OR TS=(T1D )) OR TS=(T2D)) OR TS=(MODY )) OR TS=(impaired glucose tolerance)) OR TS=(glucose intolerance)) OR TS=(insulin* depend* )) OR TS=(IDDM)) OR TS=(noninsulin* depend* )) OR TS=(NIDDM)) OR TS=(noninsulin dependent)) OR TS=(non insulindependent)) OR TS=(Noninsulindependent)) OR TS=(Insulin Resistance)) OR TS=(Glucosuria)) OR TS=(insulin sensitivity) and Preprint Citation Index (Exclude – Database) | 1711885 | 08-jan-24 |
| #1 | ((((((((((((((TS=(Light Pollution)) OR TS=(Artificial Light at Night)) OR TS=(Light at Night)) OR TS=(Night Light)) OR TS=(Nighttime Light)) OR TS=(Environmental Light*)) OR TS=(Domestic Light)) OR TS=(Artificial Night Lighting)) OR TS=(Skyglow)) OR TS=(Artificial Night Sky Brightness)) OR TS=(Light Trespass)) OR TS=(Blue Light*)) OR TS=(Light Emitting Diode*)) OR TS=(Light Exposure)) OR TS=(Glare*) and Preprint Citation Index (Exclude – Database)  ((((((((((((((TS=(Light Pollution)) OR TS=(Artificial Light at Night)) OR TS=(Light at Night)) OR TS=(Night Light)) OR TS=(Nighttime Light)) OR TS=(Environmental Light*)) OR TS=(Domestic Light)) OR TS=(Artificial Night Lighting)) OR TS=(Skyglow)) OR TS=(Artificial Night Sky Brightness)) OR TS=(Light Trespass)) OR TS=(Blue Light*)) OR TS=(Light Emitting Diode*)) OR TS=(Light Exposure)) OR TS=(Glare*) and Preprint Citation Index (Exclude – Database) | 782489 | 08-jan-24 |

**Table 1C. Embase search**

| Search | Query | Results | Date |
| --- | --- | --- | --- |
| #18 | #3 AND #6 AND #17 | 187 | 08-jan-24 |
| #17 | #9 OR #12 OR #15 OR #16 | 6,381,749 | 08-jan-24 |
| #16 | ‘observational’:ab,ti | 432,772 | 08-jan-24 |
| #15 | #13 OR #14 | 1,835,337 | 08-jan-24 |
| #14 | 'cross sectional':ab,ti OR ‘prevalence’:ab,ti OR ‘transversal’:ab,ti | 1,721,467 | 08-jan-24 |
| #13 | 'cross-sectional studies'/exp | 602,261 | 08-jan-24 |
| #12 | #10 OR #11 | 978,644 | 08-jan-24 |
| #11 | ‘case*’:ab,ti AND ‘control*’:ab,ti | 909,267 | 08-jan-24 |
| #10 | 'case-control studies'/exp | 228,433 | 08-jan-24 |
| #9 | #7 OR #8 | 4,092,932 | 08-jan-24 |
| #8 | cohort*:ab,ti | 1,513,988 | 08-jan-24 |
| #7 | 'cohort studies'/exp OR 'longitudinal study'/exp OR 'prospective study'/exp OR 'follow up study'/exp OR 'controlled clinical trial':it | 3,615,294 | 08-jan-24 |
| #6 | #4 OR #5 | 1,656,840 | 08-jan-24 |
| #5 | 'diabet*':ab,ti OR 'dm':ab,ti OR 't1dm':ab,ti OR 't2dm':ab,ti OR 't1d':ab,ti OR 't2d':ab,ti OR 'mody':ab,ti OR 'impaired glucose tolerance':ab,ti OR 'glucose intolerance':ab,ti OR 'insulin* depend* ':ab,ti OR 'iddm':ab,ti OR 'non insulin* depend* ':ab,ti OR 'niddm':ab,ti OR 'noninsulin dependent':ab,ti OR 'non insulindependent':ab,ti OR 'noninsulindependent':ab,ti OR 'insulin resistance':ab,ti OR 'glucosuria':ab,ti OR 'insulin sensitivity':ab,ti | 1,364,510 | 08-jan-24 |
| #4 | 'diabetes mellitus'/exp OR 'insulin resistance'/exp | 1,380,069 | 08-jan-24 |
| #3 | #1 OR #2 | 43,863 | 08-jan-24 |
| #2 | 'light pollution':ab,ti OR 'artificial light at night':ab,ti OR 'light at night':ab,ti OR 'night light':ab,ti OR 'nighttime light':ab,ti OR 'environmental light*':ab,ti OR 'domestic light':ab,ti OR 'artificial night lighting':ab,ti OR 'skyglow':ab,ti OR 'artificial night sky brightness':ab,ti OR 'light trespass':ab,ti OR 'blue light*':ab,ti OR 'light emitting diode*':ab,ti OR 'light exposure':ab,ti OR 'glare*':ab,ti | 43,791 | 08-jan-24 |
| #1 | 'light pollution'/exp | 485 | 08-jan-24 |

**Table 1D.** **Scopus search**

| Search | Query | Results | Date |
| --- | --- | --- | --- |
| #1 | ( TITLE-ABS-KEY ( light AND pollution ) OR TITLE-ABS-KEY ( artificial AND light AND at AND night ) OR TITLE-ABS-KEY ( light AND at AND night ) OR TITLE-ABS-KEY ( night AND light ) OR TITLE-ABS-KEY ( nighttime AND light ) OR TITLE-ABS-KEY ( environmental AND light* ) OR TITLE-ABS-KEY ( domestic AND light ) OR TITLE-ABS-KEY ( artificial AND night AND lighting ) OR TITLE-ABS-KEY ( skyglow ) OR TITLE-ABS-KEY ( artificial AND night AND sky AND brightness ) OR TITLE-ABS-KEY ( light AND trespass ) OR TITLE-ABS-KEY ( blue AND light* ) OR TITLE-ABS-KEY ( light AND emitting AND diode* ) OR TITLE-ABS-KEY ( light AND exposure ) OR TITLE-ABS-KEY ( glare* ) ) AND ( TITLE-ABS-KEY ( diabet* ) OR TITLE-ABS-KEY ( dm ) OR TITLE-ABS-KEY ( t1dm ) OR TITLE-ABS-KEY ( t2dm ) OR TITLE-ABS-KEY ( t1d ) OR TITLE-ABS-KEY ( t2d ) OR TITLE-ABS-KEY ( mody ) OR TITLE-ABS-KEY ( impaired AND glucose AND tolerance ) OR TITLE-ABS-KEY ( glucose AND intolerance ) OR TITLE-ABS-KEY ( insulin* AND depend* ) OR TITLE-ABS-KEY ( iddm ) OR TITLE-ABS-KEY ( non AND insulin* AND depend* ) OR TITLE-ABS-KEY ( niddm ) OR TITLE-ABS-KEY ( noninsulin AND dependent ) OR TITLE-ABS-KEY ( non AND insulindependent ) OR TITLE-ABS-KEY ( noninsulindependent ) OR TITLE-ABS-KEY ( insulin AND resistance ) OR TITLE-ABS-KEY ( glucosuria ) OR TITLE-ABS-KEY ( insulin AND sensitivity ) ) AND ( ( TITLE-ABS-KEY ( longitudinal ) OR TITLE-ABS-KEY ( prospective ) OR TITLE-ABS-KEY ( follow AND up ) OR TITLE-ABS-KEY ( cohort* ) ) OR ( TITLE-ABS-KEY ( case* AND control* ) ) OR ( ( TITLE-ABS-KEY ( cross-sectional ) OR TITLE-ABS-KEY ( prevalence ) OR TITLE-ABS-KEY ( transversal ) ) ) OR ( TITLE-ABS-KEY ( observational ) ) ) | 740 | 08-jan-24 |

**Table 1E.** **CINAHL search**

| Search | Query | Results | Date |
| --- | --- | --- | --- |
| S30 | S24 AND S28 AND S29 | 29 | 08-jan-24 |
| S29 | S23 OR S25 OR S26 OR S27 | 5,150,715 | 08-jan-24 |
| S28 | S1 OR S4 | 150,096 | 08-jan-24 |
| S27 | S17 OR S20 | 1,993,695 | 08-jan-24 |
| S26 | S13 OR S16 | 1,028,395 | 08-jan-24 |
| S25 | S9 OR S12 | 2,390,810 | 08-jan-24 |
| S24 | S5 OR S8 | 700,365 | 08-jan-24 |
| S23 | S21 OR S22 | 421,416 | 08-jan-24 |
| S22 | AB Observational | 373,791 | 08-jan-24 |
| S21 | Tl Observational | 85,860 | 08-jan-24 |
| S20 | 818 OR 819 | 1,865,434 | 08-jan-24 |
| S19 | AB cross-sectional OR AB prevalence OR AB transversal | 1,752,978 | 08-jan-24 |
| S18 | Tl cross-sectional OR Tl prevalence OR Tl transversal | 399,897 | 08-jan-24 |
| S17 | SU Cross-Sectional Studies | 535,379 | 08-jan-24 |
| S16 | S14 OR S15 | 848,585 | 08-jan-24 |
| S15 | AB Case* and control* | 821,063 | 08-jan-24 |
| S14 | Tl Case* and control* | 78,194 | 08-jan-24 |
| S13 | SU case-control studies | 297,684 | 08-jan-24 |
| S12 | S10 OR S11 | 1,290,024 | 08-jan-24 |
| S11 | AB Cohort* | 1,181,090 | 08-jan-24 |
| S10 | Tl Cohort* | 284,396 | 08-jan-24 |
| S9 | SU cohort studies OR SU longitudinal study OR SU prospective study OR SU follow up study | 1,486,579 | 08-jan-24 |
| S8 | 86 OR 87 | 682,057 | 08-jan-24 |
| S7 | AB diabet* OR AB DM ORAB T1DM ORAB T2DM OR AB T1 D OR AB T2D OR AB MODY OR AB impaired glucose tolerance OR AB glucose intolerance OR AB insulin* depend* OR AB IDDM OR AB non insulin* depend* OR AB NIDDM ORAB noninsulin dependent ORAB non insulindependent OR AB Noninsulindependent OR AB Insulin Resistance OR AB Glucosuria OR AB insulin sensitivity | 594,838 | 08-jan-24 |
| S6 | Tl diabet* OR Tl DM OR Tl T1 OM OR Tl T2DM OR Tl T1D OR Tl T2D OR Tl MODY OR Tl impaired glucose tolerance OR Tl glucose intolerance OR Tl insulin* depend* OR Tl IDDM OR Tl non insulin* depend* OR Tl NIDDM OR Tl noninsulin dependent OR Tl non insulindependent OR Tl Noninsulindependent OR Tl Insulin Resistance OR Tl Glucosuria OR Tl insulin sensitivity | 349,309 | 08-jan-24 |
| S5 | SU diabetes mellitus OR SU Insulin Resistance | 423,741 | 08-jan-24 |
| S4 | S2 OR S3 | 149,866 | 08-jan-24 |
| S3 | AB AB Light Pollution OR AB Artificial Light at Nigh OR AB Light at Night OR AB Night Light OR AB Nighttime Light OR AB Environmental Light* OR AB Domestic Light OR AB Artificial Night Lighting OR AB Skyglow OR AB Artificial Night Sky Brightness OR AB Light Trespass OR AB Blue Light* OR AB Light Emitting Diode* OR AB Light Exposure OR AB Glare* | 136,523 | 08-jan-24 |
| S2 | Tl Light Pollution OR Tl Artificial Light at Night OR Tl Light at Night OR Tl Night Light OR Tl Nighttime Light OR Tl Environmental Light* OR Tl Domestic Light OR Tl Artificial Night Lighting OR Tl Skyglow OR Tl Artificial Night Sky Brightness OR Tl Light Trespass OR Tl Blue Light* OR Tl Light Emitting Diode* OR Tl Light Exposure OR Tl Glare* | 33,680 | 08-jan-24 |
| S1 | SU Light Pollution | 1,489 | 08-jan-24 |

Appendix Table 2: Excluded studies and reasons for their exclusion after full texts assessment with additional reference

| Rank | First Author | Year | Title | Reason for Exclusion |
| --- | --- | --- | --- | --- |
| 1 | Anjum, B. | 2015 | Light at night (LAN) and rotating night shift associated with metabolic risk factors for type 2 diabetes | Conference papers |
| 2 | Anjum, B. | 2014 | Light at night (LAN) as a risk of coronary artery disease (CAD) and type 2 diabetes in rotating night shift nursing professionals | Conference papers |
| 3 | Obayashi, K. | 2019 | Nighttime light exposure and the incidence of diabetes mellitus: a longitudinal study of the HEIJO-KYO cohort | Conference papers |
| 4 | Barker, A. | 2018 | Physical activity, sedentary time, TV viewing, physical fitness and cardiovascular disease risk in adolescents: The HELENA study | Not relevant exposure of light pollution |
| 5 | Benedito-Silva, A. | 2020 | Association between light exposure and metabolic syndrome in a rural Brazilian town | No relevant diabetes outcome |
| 6 | Borroni, E. | 2023 | Metabolomic profiles in night shift workers: A cross-sectional study on hospital female nurses | No relevant diabetes outcome |
| 7 | Deng, N. | 2018 | The Relationship Between Shift Work and Men's Health | Not relevant exposure of light pollution |
| 8 | Esquirol, Y. | 2009 | Shift Work and Metabolic Syndrome: Respective Impacts of Job Strain, Physical Activity, and Dietary Rhythms | Not relevant exposure of light pollution |
| 9 | Fonken, L. | 2011 | Illuminating the deleterious effects of light at night | No relevant diabetes outcome |
| 10 | Gil-Lozano, M. | 2016 | Short-term sleep deprivation with nocturnal light exposure alters time-dependent glucagon-like peptide-1 and insulin secretion in male volunteers | Not correspond to the definition of light pollution |
| 11 | Harmsen, J. | 2022 | The influence of bright and dim light on substrate metabolism, energy expenditure and thermoregulation in insulin-resistant individuals depends on time of day | Not correspond to the definition of light pollution |
| 12 | Knutson, K. | 2017 | Association Between Sleep Timing, Obesity, Diabetes: The Hispanic Community Health Study/Study of Latinos (HCHS/SOL) Cohort Study | Not relevant exposure of light pollution |
| 13 | Lewis, P. | 2021 | Perinatal photoperiod associations with diabetes and chronotype prevalence in a cross-sectional study of the UK Biobank | Not correspond to the definition of light pollution |
| 14 | Lin, J. | 2023 | Association of time spent in outdoor light and genetic susceptibility with the risk of type 2 diabetes | Not correspond to the definition of light pollution |
| 15 | Obayashi, K | 2022 | Associations between indoor light pollution and unhealthy outcomes in 2,947 adults: Cross-sectional analysis in the HEIJO-KYO cohort | No relevant diabetes outcome |
| 16 | Shah, A. | 2022 | Is Shift Work Sleep Disorder a Risk Factor for Metabolic Syndrome and Its Components? A Systematic Review of Cross-Sectional Studies | Not relevant exposure of light pollution |
| 17 | Sorensen, T | 2020 | Is night-time light intensity associated with cardiovascular disease risk factors among adults in early-stage urbanisation in South India? A cross-sectional study of the Andhra Pradesh Children and Parents Study | No relevant diabetes outcome |
| 18 | Tappo, S. | 2022 | Spatial association of socio-demographic, environmental factors and prevalence of diabetes mellitus in middle-aged and elderly people in Thailand | Not relevant exposure of light pollution |
| 19 | Tsc, S. | 2014 | Preliminary results of shift work and cardiovascular risk factors: analysing baseline data of a prospective night shift worker cohort in Shenzhen, China | No relevant diabetes outcome |
| 20 | Versteeg, R. | 2016 | Acute effect of ambient light intensity on glucose and lipid metabolism and appetite in healthy humans and obese patients with type 2 diabetes | Not correspond to the definition of light pollution |
| 21 | Xu, Y. | 2022 | Exposure to bedroom light pollution and cardiometabolic risk: A cohort study from Chinese young adults | No relevant diabetes outcome |
| 22 | Xu, Y. | 2024 | Association of light at night with cardiometabolic disease: A systematic review and meta-analysis | Study type was not a observation study |
| 23 | Yi, W. | 2023 | Association of outdoor artificial light at night with metabolic syndrome and the modifying effect of tree and grass cover | No relevant diabetes outcome |

**Additional reference**

1. Anjum B, Verma NS, Tiwari S, et al. Light at night (LAN) and rotating night shift associated with metabolic risk factors for type 2 diabetes. *International Journal of Diabetes in Developing Countries* 2015;35(4):S539. doi: doi:10.1007/s13410-016-0469-6

2. Anjum B, Verma NS, Tiwari S, et al. Light at night (LAN) as a risk of coronary artery disease (CAD) and type 2 diabetes in rotating night shift nursing professionals. *Endocrine Practice* 2014;20(1):10A-11A. doi: doi:

3. Obayashi K, Yamagami Y, Kurumatani N, et al. NIGHTTIME LIGHT EXPOSURE AND THE INCIDENCE OF DIABETES MELLITUS: A LONGITUDINAL STUDY OF THE HEIJO-KYO COHORT. *Sleep Medicine* 2019;64:S282-S82. doi: doi:

4. Barker AR, Gracia-Marco L, Ruiz JR, et al. Physical activity, sedentary time, TV viewing, physical fitness and cardiovascular disease risk in adolescents: The HELENA study. *International Journal of Cardiology* 2018;254:303-09. doi: doi:10.1016/j.ijcard.2017.11.080

5. Benedito-Silva AA, Evans S, Mendes JV, et al. Association between light exposure and metabolic syndrome in a rural Brazilian town. *PLoS ONE* 2020;15(9) doi: doi:10.1371/journal.pone.0238772

6. Borroni E, Frigerio G, Polledri E, et al. Metabolomic profiles in night shift workers: A cross-sectional study on hospital female nurses. *Frontiers in Public Health* 2023;11 doi: doi:10.3389/fpubh.2023.1082074

7. Deng N, Kohn TP, Lipshultz LI, et al. The Relationship Between Shift Work and Men's Health. *Sexual Medicine Reviews* 2018;6(3):446-56. doi: doi:10.1016/j.sxmr.2017.11.009

8. Esquirol Y, Bongard V, Mabile L, et al. Shift Work and Metabolic Syndrome: Respective Impacts of Job Strain, Physical Activity, and Dietary Rhythms. *Chronobiology International* 2009;26(3):544-59. doi: doi:10.1080/07420520902821176

9. Fonken LK, Nelson RJ. Illuminating the deleterious effects of light at night. *F1000 Medicine Reports* 2011;3(1) doi: doi:10.3410/M3-18

10. Gil-Lozano M, Hunter PM, Behan LA, et al. Short-term sleep deprivation with nocturnal light exposure alters time-dependent glucagon-like peptide-1 and insulin secretion in male volunteers. *Am J Physiol Endocrinol Metab* 2016;310(1):E41-50. doi: doi:10.1152/ajpendo.00298.2015

11. Harmsen JF, Wefers J, Doligkeit D, et al. The influence of bright and dim light on substrate metabolism, energy expenditure and thermoregulation in insulin-resistant individuals depends on time of day. *Diabetologia* 2022;65(4):721-32. doi: doi:10.1007/s00125-021-05643-9

12. Knutson KL, Wu D, Patel SR, et al. Association Between Sleep Timing, Obesity, Diabetes: The Hispanic Community Health Study/Study of Latinos (HCHS/SOL) Cohort Study. *Sleep* 2017;40(4) doi: doi:10.1093/sleep/zsx014

13. Lewis P, Morfeld P, Mohren J, et al. Perinatal photoperiod associations with diabetes and chronotype prevalence in a cross-sectional study of the UK Biobank. *Chronobiol Int* 2021;38(3):343-59. doi: doi:10.1080/07420528.2020.1849254

14. Lin J, Yang H, Zhang Y, et al. Association of time spent in outdoor light and genetic susceptibility with the risk of type 2 diabetes. *Science of the Total Environment* 2023;888 doi: doi:10.1016/j.scitotenv.2023.164253

15. Obayashi K, Tai Y, Yamagami Y, et al. Associations between indoor light pollution and unhealthy outcomes in 2,947 adults: Cross-sectional analysis in the HEIJO-KYO cohort. *Environmental Research* 2022;215 doi: doi:10.1016/j.envres.2022.114350

16. Shah A, Turkistani A, Luenam K, et al. Is Shift Work Sleep Disorder a Risk Factor for Metabolic Syndrome and Its Components? A Systematic Review of Cross-Sectional Studies. *Metabolic Syndrome and Related Disorders* 2022;20(1):1-10. doi: doi:10.1089/met.2021.0070

17. Sorensen TB, Wilson R, Gregson J, et al. Is night-time light intensity associated with cardiovascular disease risk factors among adults in early-stage urbanisation in South India? A cross-sectional study of the Andhra Pradesh Children and Parents Study. *Bmj Open* 2020;10(11) doi: doi:10.1136/bmjopen-2019-036213

18. Tappo S, Laohasiriwong W, Puttanapong N. Spatial association of socio-demographic, environmental factors and prevalence of diabetes mellitus in middle-aged and elderly people in Thailand. *Geospatial health* 2022;17(2) doi: doi:10.4081/gh.2022.1091

19. Tsc SLA, Wang F, Zhang L, et al. 0174Preliminary results of shift work and cardiovascular risk factors: analysing baseline data of a prospective night shift worker cohort in Shenzhen, China. *Occupational & Environmental Medicine* 2014;71:A81-2. doi: doi:10.1136/oemed-2014-102362.254

20. Versteeg RI, Stenvers DJ, Fliers E, et al. Acute effect of ambient light intensity on glucose and lipid metabolism and appetite in healthy humans and obese patients with type 2 diabetes. *Proceedings of the Nutrition Society* 2016;75:E13. doi: doi:10.1017/S0029665115004462

21. Xu YX, Yu Y, Huang Y, et al. Exposure to bedroom light pollution and cardiometabolic risk: A cohort study from Chinese young adults. *Environ Pollut* 2022;294:118628. doi: doi:10.1016/j.envpol.2021.118628

22. Xu YX, Zhang JH, Ding WQ. Association of light at night with cardiometabolic disease: A systematic review and meta-analysis. *Environ Pollut* 2024;342:123130. doi: doi:10.1016/j.envpol.2023.123130

23. Yi W, Wang W, Xu Z, et al. Association of outdoor artificial light at night with metabolic syndrome and the modifying effect of tree and grass cover. *Ecotoxicol Environ Saf* 2023;264:115452. doi: doi:10.1016/j.ecoenv.2023.115452

Appendix Table 3: Quality assessment of the included studies

Table 3A: Newcastle-Ottawa Scale (NOS) for assessing the risk of bias in the cohort of this review

| **Domain** | **Item** | **Description** | **Score (star)** |
| --- | --- | --- | --- |
| Selection | Representativeness of the exposed cohort | Exposed population was from the general population sampled randomly or close to the general population, which could truly or to some extent reflect the exposure factors (light pollution) | * |
|  | Selection of the non-exposed cohort | Drawn from the same community as the exposed cohort | * |
|  | Ascertainment of exposure | Describe exposure factors (light pollution) objectively | * |
|  | Interest outcome | Demonstration that outcome of interest was not present at start of study | * |
| Comparability | Comparability of cohorts on the basis of the design or analysis | A. The most important influencing factors (including but not limited to age, gender, residence, smoking, socioeconomic status) were controlled by different calculation models in the study. | * |
|  |  | B. Different computational models were used to control for other important confounding factors in the study (e.g., physical activity, body mass index, history of diseases, medications, health behaviors, body mass index, etc.) | * |
| Outcome | Assessment of outcome | Independent blind assessment or record linkage | * |
|  | Follow-up for outcome | Select 5-10 years follow up period for outcome of interest | * |
|  | Adequacy of follow up of cohorts | Bias due to loss to follow-up of less than 20% was acceptable | * |

- ≥7* are high quality studies, 5*-6* are medium quality studies, ≤4* are low quality studies

Table 3B: Quality assessment of the included cohort studies based on the NOS

| Study ID | Selection | | | | Comparability | Outcome | | | Total  Score  (Star) |
| --- | --- | --- | --- | --- | --- | --- | --- | --- | --- |
|  | Representativeness of the exposed cohort | Selection of the  Non-exposed cohort | Ascertainment of exposure | Interest outcome | Comparability of cohorts | Assessment of outcome | Follow-up for outcomes | Adequacy of follow up of cohorts |  |
| Obayashi, K,2020 |  | * | * | * | ** | * |  |  | 6* |
| Wang, C,2023 | * | * |  | * | ** | * | * |  | 7* |
| Xu, Z,2023 | * | * | * | * | ** | * | * |  | 8* |

Table 3C: Reason for assessment of the included cohort studies based on the Newcastle-Ottawa Scale

| Study ID | Selection | | | | | Comparability | Outcome | | |
| --- | --- | --- | --- | --- | --- | --- | --- | --- | --- |
|  | Representativeness of the exposed cohort | Selection of the  Non-exposed cohort | Ascertainment of exposure | Interest outcome | Comparability of cohorts | | Assessment of outcome | Follow-up for outcomes | Adequacy of follow up of cohorts |
| Obayashi, K,2020 | The population is from the HEIJO-KYO cohort, and we believe that the population is from a single source and is all elderly, which is not representative of the overall population. | The control and exposed groups were from the same area. | Measurement of light pollution is described in the methodology section. | All participants were assessed for diabetes prior to enrolment. | Adjusting for confounders using Poisson regression models. | | The patient's venous blood was collected to determine HbA1c in conjunction with the history for evaluation. | Follow-up lasts 42 months. | not report |
| Wang, C,2023 | The population was obtained from the UK Biobank and was selected from people aged 40-69 years old. | The control and exposed groups were from the same area. | Severity of exposure determined only by means of questionnaire. | All participants were assessed for diabetes prior to enrolment. | Three different models were developed to adjust for confounders. | | Assessment using objective hospitalisation information. | Median follow-up period of 13.04 years. | not report |
| Xu, Z,2023 | The population was obtained from the UK Biobank and was selected from people aged 37-73 years. | The control and exposed groups were from the same area. | Measurement of light pollution is described in the methodology section. | All participants were assessed for diabetes prior to enrolment. | Stratification of different confounders using Cox modelling. | | Assessment using objective hospitalisation information. | Follow-up lasts 14 years. | not report |

Table 3D: Agency for Healthcare Research and Quality (AHRQ) for assessing the risk of bias in the Cross-sectional studies of this review

| **Domain** | **Items** | **Assessment** |
| --- | --- | --- |
| Selection bias | 1.Define the source of information (survey, record review) | Yes/Unclear/No |
|  | 2.List inclusion and exclusion criteria for exposed and unexposed subjects (cases and controls) or refer to previous publications | Yes/Unclear/No |
|  | 3.Indicate time period used for identifying patients | Yes/Unclear/No |
|  | 4.Indicate whether or not subjects were consecutive if not population-based | Yes/Unclear/No |
| Implementation of bias | 5.Explain any patient exclusions from analysis | Yes/Unclear/No |
|  | 6.Describe how confounding was assessed and/or controlled | Yes/Unclear/No |
| Measurement bias | 7.If applicable, explain how missing data were handled in the analysis | Yes/Unclear/No |
| Follow-up of bias | 8.Describe any assessments undertaken for quality assurance purposes (eg: test/retest of primary outcome measurements) | Yes/Unclear/No |
|  | 9.Summarize patient response rates and completeness of data collection | Yes/Unclear/No |
|  | 10.Clarify what follow-up, if any, was expected and the percentage of patients for which incomplete data or follow-up was obtained | Yes/Unclear/No |
| Reporting bias | 11.Indicate if evaluators of subjective components of study were masked to other aspects of the status of the participants | Yes/Unclear/No |

- One point for "yes" and no points for "no" and "unclear". 8-11 were classified as high quality studies, 5-7 as moderate quality studies and 0-4 as low quality studies.

Table 3E: Quality assessment of the included Cross-sectional studies based on the AHRQ

| **Study ID** | **1** | **2** | **3** | **4** | **5** | **6** | **7** | **8** | **9** | **10** | **11** | **Total**  **Score** |
| --- | --- | --- | --- | --- | --- | --- | --- | --- | --- | --- | --- | --- |
| Kim, M,2023 | yes | yes | yes | yes | unclear | yes | yes | yes | yes | no | yes | 8 |
| Obayashi, K,2014 | yes | unclear | yes | no | unclear | yes | unclear | no | unclear | no | yes | 4 |
| Zheng, R,2023 | yes | yes | yes | yes | unclear | yes | yes | no | yes | yes | yes | 9 |

Table 3F: Reason for assessment of the included Cross-sectional studies based on the AHRQ

| **Study ID** | **1** | **2** | **3** | **4** | **5** | **6** | **7** | **8** | **9** | **10** | **11** |
| --- | --- | --- | --- | --- | --- | --- | --- | --- | --- | --- | --- |
| Kim, M,2023 | The Chicago Healthy Aging Study (CHAS) is a study of a subset of participants from the Chicago Heart Association Detection Project in Industry (CHA). | The details of the grouping are given in the section on the statistical analysis. | Specific times indicated. | Describes the process of sampling from CHA. | Not report | The process of controlling for confounders is described in the Statistical analysis section. | Described in the Missing data section. | Sensitivity analyses were conducted to evaluate the stability of the results. | Described in the Missing data section. | Not report | NA |
| Obayashi, K,2014 | Sourced from HEIJO-KYO cohort. | Not report | Specific times indicated. | The sample population included was older and there was no recruitment programme. | Not report | The process of controlling for confounders is described in the Statistical analysis section. | Not report | Not report | Not report | Not report | NA |
| Zheng, R,2023 | The National Disease Surveillance Point System of Chinese Center for Disease Control and Prevention. | The details of the grouping are given in the section on the statistical analysis. | Specific times indicated. | Source of population with detailed sampling programme. | Not report | The process of controlling for confounders is described in the Statistical analysis section. | Describes the processing of Missing data | Not report | Missing data are described in the Statistical analysis section. | Missing data are described in the Statistical analysis section. | NA |

Appendix Table 4: General characteristics for the studies included in the review

| **Study ID** | **Study Population** | **geographic location** | **Study design** | **Follow-up time** | **Number of participants  (event/total)  (mean age, years)  (male,%)** | **Recruitment strategy** | **Response  rate** | **Exposure  (Type of light pollution)** | **Outcome** | **Objective** | **Conclusion** | **Funding source.** | **Conflict of interest** |
| --- | --- | --- | --- | --- | --- | --- | --- | --- | --- | --- | --- | --- | --- |
| Kim, M,2023 | 632 community-dwelling adults aged 63-84 years. | United States | cross-sectional study | NA | 552  (297/552)  (72,5)  (410,74) | inclusion criteria:Low-risk status (LR) was defined as having favorable levels of four major CVD risk factors: serum total cholesterol level <200 mg/dL and no use of cholesterol-lowering medication; systolic blood pressure (SBP) ≤120 mmHg, diastolic BP (DBP) ≤80 mmHg and no use of antihypertensive medication; no current smoking; and no history of diabetes or heart attack. | 552/632 | Indoor LAN | risk of diabetes | The objectives of this study were (1) to characterize objective light exposure patterns in a cohort of community-dwelling older adults and (2) to determine whether LAN in older age is associated with higher prevalence of individual CVD risk factors. | Habitual LAN in older age is associated with concurrent obesity, diabetes, and hypertension. | This research was supported by the National Institutes of Health National Heart, Lung, and Blood Institute (grant R01 HL089695, R01 HL090873, R01 HL021010), the National Center for Advancing Translational Sciences (UL1TR001422), the National Institute on Aging (P30AG059988), and Northwestern University Feinberg School of Medicine Ken and Ruth Davee Department of Neurology. | no conflict of interest |
| Obayashi, K,2014 | 537 community-based elderly subjects. | Japanese | cross-sectional study | NA | 513  (69,513)  (72.7±6.5)  (238,46.4) | inclusion criteria: age ≥60 years and complete records of UME and evening light measurement. | 513/537 | Indoor ELavg (evening light) | risk of diabetes | The objective of this study were to characterize whether exposure to evening light and decreased melatonin secretion are associated with diabetes. | This study in elderly individuals demonstrated that evening light exposure in home settings and UME were significantly and independently associated with diabetes. | This work was supported by Grants from the Department of Indoor Environmental Medicine, Nara Medical University; Scientific Research from the Ministry of Education, Culture, Sports, Science and Technology; Mitsui Sumitomo Insurance Welfare Foundation; Meiji Yasuda Life Foundation of Health and Welfare; Osaka Gas Group Welfare Foundation; Japan Diabetes Foundation; and the Japan Science and Technology Agency. | no conflict of interest |
| Obayashi, K,2020 | 954 elderly participants without diabetes. | Japanese | cohort study | median,3.5 years | 678  (19,678)  (70.6±6.6)  (302,44.5) | inclusion criteria: age ≥60 years and exclud diabetes | 678/954 | Indoor LAN | risk of diabetes | The aim of the present study was to evaluate a longitudinal association between LAN exposure and the incidence of diabetes in a general population. | Our findings suggest that LAN exposure increases the incidence of diabetes in a general elderly population. | This work was supported by research funding from the Department of Indoor Environmental Medicine, Nara Medical University; JSPS KAKENHI (grant numbers: 24790774, 22790567, 25860447, 25461393, 15H04776, and 10124877); Mitsui Sumitomo Insurance Welfare Foundation; Meiji Yasuda Life Foundation of Health and Welfare; Osaka Gas Group Welfare Foundation; Japan Diabetes Foundation; Daiwa Securities Health Foundation; Japan Science and Technology Agency; YKK AP Inc.; Ushio Inc.; Nara Prefecture Health Promotion Foundation; Nara Medical University Grant-in-Aid for Collaborative Research Projects; Tokyo Electric Power Company; EnviroLife Research Institute Co., Ltd.; Sekisui Chemical Co., Ltd; LIXIL Corp.; and KYOCERA Corp. | no conflict of interest |
| Wang, C,2023 | 471,686 participants without diabetes were recruited for the present study. | United Kingdom | cohort study | median, 13.04 years | 471,686  (18738/471,686)  (56.3±8.11)  (210692,44.65) | inclusion criteria:age 40-69 years and exclud diabetes excluding the participants without the information needed for the online questionnaire and those who were diagnosed with T2D before participating in the UK Biobank baseline survey | NA | Indoor blue light | risk of diabetes | The present study aimed to investigate the relationship between blue light exposure and T2D incidence and whether it is affected by sleep duration, physical activity, outdoor activity time, and genetic susceptibility. | The present study showed that blue light exposure is associated with a greater risk of T2D independent of classical T2D risk factors. | This research was supported by the National Natural Science Foundation of China (82,273,613). | no conflict of interest |
| Xu, Z,2023 | 283,374 participants to be included in our analysis. | United Kingdom | cohort study | 14 years | 283,374  (7775)  (55.8±8.10)  (136,510,48.2) | inclusion criteria: a.37-73 years; b.without any form of T2DM; c.with available geolocation information and nightlight exposure; d.excluding cases with missing values on confounders | NA | Outdoor LAN | risk of diabetes | The present study aimed to investigate the relationship between long-term LAN exposure on T2DM incidence. | Exposuring to residential outdoor LAN may contribute to T2DM risk and low sleep quality. | This work was supported by the China National Key Research and Development Program (2018YFE0115300) and the State Scholarship Fund of China Scholarship Council (202006015008, 202006015015). | no conflict of interest |
| Zheng, R,2023 | 98,658 Chinese adults responded and participated in the survey. | China | cross-sectional study | NA | 98,658  (5525,98658)  (NA)  (54143,54.9) | NA | 98658/109023 | Outdoor LAN | risk of diabetes | Our aim was to estimate the associations of chronic exposure to outdoor LAN with glucose homoeostasis markers and diabetes prevalence based on a national and cross-sectional survey of the general population in China. | Our findings contribute to the growing evidence that LAN is detrimental to health and point to outdoor LAN as a potential novel risk factor for diabetes. | This work was supported by the grants from the National Natural Science Foundation of China (81870560, 8208810, 81970691, 81941017, 81770842, 81970706, 82022011, 82070880), the Shanghai Shenkang Hospital Development Center (SHDC12019101, SHDC2020CR1001A, SHDC2020CR3064B), the Shanghai Municipal Government (20Y11905100), Shanghai Jiaotong University School of Medicine (DLY201801) and the Ruijin Hospital (2018CR002). | no conflict of interest |

Appendix Table 5: Explanation of light pollution in the studies included in the review

| **Study ID** | **Definition of light pollution** |
| --- | --- |
| Kim, M,2023 | The least active 5-h period (L5) computed from minute-wise averages across 24-h periods. |
| Obayashi, K,2014 | Evening light (ELavg), the average light intensity during the 4 h prior to bedtime,night-time light (NLavg), the average light intensity during the in-bed period. |
| Obayashi, K,2020 | LAN was defined as the average light intensity recorded between bedtimes and rise times over two consecutive nights. |
| Wang, C,2023 | Blue light exposure level. Incould: watching TV, using the computer, playing computer games. |
| Xu, Z,2023 | Annual outdoor light at night. |
| Zheng, R,2023 | Used the high dynamic range data throughout the year 2010 downloaded from the NGDC. The mean night-time radiance of the outdoor LAN for each study site, which was either a district in a city or a county in a rural area, was calculated and participants living within each study site were assigned the same mean radiance of the outdoor LAN at that study site. |

**Appendix Table 6:** Exposure assessment in the studies included in the review

| **Study ID** | **Exposure location** | **Exposure indicator(s)** | **Data source** | **Timepoint of data collection** | **Summary statistics of exposure intensity** |
| --- | --- | --- | --- | --- | --- |
| Kim, M,2023 | United States, community dwelling, indoor | L5: midpoint of the least active 5-h period computed from minute-wise averages across 24-h periods. After inspecting the distribution of L5 light values (i.e. zero-inflated), a decision was made to categorize L5 light as 0 or >0. Participants with 0 light during the 5-h nadir (L5) were categorized as having “No-LAN,” while those with >0 light during L5 were categorized as having “LAN.” | wrist actigraphy monitor: Actiwatch-L | post-treatment | NA |
| Obayashi, K,2014 | Japan, community dwelling, indoor | Quartiles and medians of mean light intensity during the 4 hours before bedtime to differentiate exposure intensity. | wrist light meter (Actiwatch 2; Respironics Inc., Pittsburgh, PA) | post-treatment | 25.4 lux (IQR, 17.5–37.6) |
| Obayashi, K,2020 | Japan, indoor | LAN avg ≥ 5 lux is considered to have light pollution exposure. | portable light meter (LX-28SD; Sato Shouji Inc., Kanagawa, Japan) | During follow-up period | NA |
| Wang, C,2023 | United Kingdom, indoor | Participants' blue light exposure data were collected via an questionnaire, a total score of 0 or 1 represented mild blue light exposure, 2 represented moderate blue light exposure, and 3 represented heavy blue light exposure. | online Health Work questionnaire | During follow-up period | NA |
| Xu, Z,2023 | United Kingdom, outdoor | Confirmation of the intensity of exposure through the light intensity of the annual outdoor LAN average of the 500 m2 buffer zone for each participant at their follow-up time. | Defense Meteorological Satellite Program Operational Linescan System (DMSP) and Suo mi National Polar-orbiting Partnership Visible Infrared Imaging Radiometer Suite (NPP-VIIRS) | During follow-up period | 8.00nW/cm2/sr (Range, 0–155.42) |
| Zheng, R,2023 | China, outdoor | The intensity of exposure was confirmed by participants residing within each study site being assigned to the same average outdoor LAN radiance for that study site. | Defense Meteorological Satellite Program (DMSP) | post-treatment | 7.9nW/cm2/sr, (IQR,2.8,26.8) |

Appendix Table 7: Statistical approaches in the studies included in the review

| **Study ID** | **Statistical model** | **Stratified analysis** | **subgroup analysis** | **Adjustment for confounders or factors** | **Sensitivity analysis** | **Increment unit** |
| --- | --- | --- | --- | --- | --- | --- |
| Kim, M,2023 | Multivariable model | CVD risk | Low-LAN; High-LAN | age, sex, race, season. | yes(age, sex, race, season, WASO, SE, sleep onset, sleep midpoint, sleep offset, TST, time in bed, mean activity count during the L5 light period, mean 24-h activity count, M10 activity, L5 activity, IS activity, IV activity, RA of activity, and timing of M10 and L5 activity) | NA |
| Obayashi, K,2014 | Multivariate model | NA | NA | gender, BMI, duration in bed and Nlavg. | NA | Per two-unit IQR increment |
| Obayashi, K,2020 | Poisson regression models | NA | NA | age, gender, current smoking status, alcohol consumption, education, household income, BMI, hypertension, caloric intake, daytime physical activity, bedtime, rise time, daytime light exposure, actigraphic TST,SE. | yes(Not elaborated) | NA |
| Wang, C,2023 | Cox proportional hazards (CPH) models | physical activity level; outdoor activity time in the summer and winter (>1 h); sleep quality; T2D-PRS | Moderate light;  Heavylight | age, sex, ethnicity, education level, income level, Townsend index, smoking, alcohol use, healthy diet, body mass index, physical activity, hypertensive disorders, sleep quality score, time spent outdoors in the summer, time spent outdoors in the winter, cardiovascular disease, and cancer. | yes(age, education, BMI, smoking, alcohol consumption, income, hypertensive disorders, Townsend, healthy diet index, CVD, and cancer) | NA |
| Xu, Z,2023 | Time-varying Cox proportional hazard model | polygenic risk score; shift work | Quarter 2; Quarter 3; Quarter 4 | age, sex, ethnicity, region, education, economic activity, household income, income score, housing score, shift work, smoking status, drink frequency, physical activity, sedentary time, health diet score, PM2.5, NO2, night noise, PRS, a higher score means higher genetic predisposition, population density in home geolocation with a buffer 1 km2 | yes(missing covariate values with 10 data, T2DM within the first 2 years of follow-up, extreme exposure, air pollution, ) | Per an-unit IQR increment |
| Zheng, R,2023 | Multivariable models | NA | Quintile 2; Quintile 3; Quintile 4; Quintile 5 | age, sex, education, smoking status, drinking status, physical activity, family history of diabetes, household income, urban/rural living, taking antihypertensive medications, taking lipid-lowering medications, BMI. | NA | Per an-unit IQR increment |

Appendix Table 8: Associations between exposure to light pollution and diabetes outcome

| **Study ID** | **Health outcome** | **Stratification** | **Exposure classfication** | **Number of cases** | **Type of Effect size** | **Effect size  [95% CI]** | **P value** |
| --- | --- | --- | --- | --- | --- | --- | --- |
| Kim, M,2023 | risk of diabetes | Overall | No LAN | 25/255 | OR | 1 | Ref |
|  |  |  | LAN | 53/297 | OR | 2[1.19-3.43] | 0.01 |
|  |  |  | Low-LAN | 25/149 | OR | 1.87[1.01-3.45] | 0.045 |
|  |  |  | High-LAN | 28/148 | OR | 2.15[1.17-3.97] | 0.014 |
|  |  | Low CVD risk | No LAN | 13/73 | OR | 1 | Ref |
|  |  |  | LAN | 33/84 | OR | 1.13[0.36-3.67] | 0.835 |
|  |  | Not Low CVD risk | No LAN | 55/182 | OR | 1 | Ref |
|  |  |  | LAN | 88/213 | OR | 2.28[1.26-4.26] | 0.008 |
| Obayashi, K,2014 | risk of diabetes | Overall | Elavg | 69/513 | OR | 1.72[1.12-2.64] | 0.01 |
|  |  |  | Continuous (25–75th percentiles) | NA | OR | 1.51[1.08-2.11] | NA |
| Obayashi, K,2020 | risk of diabetes | Overall | Dark avg < 5 lux | 10/550 | RR | 1 | Ref |
|  |  |  | LAN avg ≥ 5 lux | 9/128 | RR | 3.17[1.32-7.63] | 0.01 |
| Wang, C,2023 | risk of diabetes | Overall | Light | 3183/115703 | HR | 1 | Ref |
|  |  |  | Moderate | 11734/281763 | HR | 1.12[1.07-1.16] | <0.05 |
|  |  |  | Heavy | 3821/74850 | HR | 1.19[1.14-1.25] | <0.05 |
|  |  | vigorous metabolic equivalent task | heavy vs light | NA | HR | 1.39[1.25-1.54] | NA |
|  |  | time spent outdoors in summer < 3H | heavy vs light | NA | HR | 1.14[1.07-1.22] | NA |
|  |  | time spent outdoors in winter < 1H | heavy vs light | NA | HR | 1.13[1.06-1.21] | NA |
|  |  | Healthy sleep quality | heavy vs light | NA | HR | 1.23[1.10-1-36] | NA |
|  |  | Healthy sleep quality | Moderate vs light | NA | HR | 1.15[1.06-1.26] | NA |
| Xu, Z,2023 | risk of diabetes | Overall | Quarter 1 | 1584/70,844 | HR | 1 | Ref |
|  |  |  | Quarter 2 | 1866/70,843 | HR | 1.05[0.97-1.14] | 0.248 |
|  |  |  | Quarter 3 | 2058/70,824 | HR | 1.11[1.01-1.21] | 0.031 |
|  |  |  | Quarter 4 | 2267/70,863 | HR | 1.14[1.02-1.27] | 0.023 |
|  |  |  | Continuous (per IQR increase) | NA | HR | 1.05[1.01-1.09] | 0.023 |
|  |  |  | Continuous (per 10-unit increase) | NA | HR | 1.04[1.01-1.08] | 0.023 |
|  |  | Intermediate PRS | Quarter 2 VS Quarter 1 | NA | HR | -0.04[-0.31-0.23] | NA |
|  |  |  | Quarter 3 VS Quarter 1 | NA | HR | -0.01[-0.28-0.27] | NA |
|  |  |  | Quarter 4 VS Quarter 1 | NA | HR | -0.12[-0.40-0.17] | NA |
|  |  | High PRS | Quarter 2 VS Quarter 1 | NA | HR | -0.19[-0.46-0.07] | NA |
|  |  |  | Quarter 3 VS Quarter 1 | NA | HR | -0.09[-0.35-0.17] | NA |
|  |  |  | Quarter 4 VS Quarter 1 | NA | HR | -0.20[-0.51-0.17] | NA |
|  |  | Shift work | Continuous (per IQR increase (11.22 nW/cm2/sr)) | NA | HR | 0.87[0.67-1.11] | NA |
| Zheng, R,2023 | risk of diabetes | Overall | Quintile 1 | NA/24151 | PR | 1 | Ref |
|  |  |  | Quintile 2 | 1239/14392 | PR | 1.07[0.86-1.33] | NA |
|  |  |  | Quintile 3 | 1292/15028 | PR | 1.01[0.87-1.29] | NA |
|  |  |  | Quintile 4 | 2032/23773 | PR | 1.15[0.91-1.45] | NA |
|  |  |  | Quintile 5 | 1812/21314 | PR | 1.28[1.03-1.60] | NA |
|  |  |  | per-quintile outdoor LAN exposure | NA | PR | 1.07[1.02- 1.12] | NA |

**Appendix Table 9: GRADE assessment for the association between greenspace and cancer related outcome**

| **Domain** | **Item** | **Assessment criteria** |
| --- | --- | --- |
| Downgrading | Risk of bias | Consideration was given to the soundness of the study cohort design, completeness of outcome reporting, and control for confounding factors |
|  | Inconsistency | The measures were heterogeneity tests for effect sizes and the absence of overlap in the reported confidence intervals |
|  | Imprecision | A small number of studies showed associations in the opposite direction to the same exposure outcome or confidence intervals that crossed clinical decision thresholds |
|  | Indirectness | Consider population, exposure and study outcome measured consistent with target population, exposure and outcome measures |
|  | Publication bias | If the number of studies was sufficient, it could be assessed by Egger's test; if the number of studies was insufficient, it could be assessed in terms of the design of each study, grant sponsorship, and reporting of results |
| Upgrading | Magnitude of association | To fit the effect size of escalation to the field of light pollution, and escalation was considered in terms of increases or decreases in reported effect sizes of 40% or more |
|  | Residual confounding | The effect of confounding factors on effect estimates was reduced rather than exaggerated can be considered for upgrading |
|  | Dose-response trend | Not relevant to the topic of this review |

Table 9A: GRADE assessment criteria

- Case-control trials initially rated "high", observational studies initially rated "low".

Table 9B: GRADE assessment for the quality of the body of evidence

| Outcome | Quality assessment | | | | | | | No. of studies | Relative effect [95% CI] | Overall quality |
| --- | --- | --- | --- | --- | --- | --- | --- | --- | --- | --- |
|  | Downgrading | | | | | Upgrading | |  |  |  |
|  | Risk of bias | Inconsistency | Imprecision | Indirectness | Publication bias | Magnitude of association | Residual confounding |  |  |  |
| Incidence of diabetes | none | none | none | yes | none | none | none | 6 | 1.20[1.15-1.25] | **⊕⊕⊕⊝** |
| Incidence of diabetes with low and moderate light pollution | none | none | none | yes | none | none | none | 4 | 1.10[1.06-1.14] | **⊕⊕⊕⊝** |
| Incidence of diabetes with heavy light pollution | none | none | none | yes | none | none | none | 4 | 1.19[1.14-1.24] | **⊕⊕⊕⊝** |
| Incidence of diabetes with Indoor light pollution | none | none | none | yes | none | none | none | 4 | 1.66[1.14-2.40] | **⊕⊕⊕⊝** |
| Incidence of diabetes with outdoor light pollution | none | none | none | none | none | none | none | 2 | 1.17[1.06-1.29] | **⊕⊕⊕⊕** |
| Incidence of diabetes in Europe and America | none | none | none | yes | none | none | none | 3 | 1.19[1.14-1.24] | **⊕⊕⊕⊝** |
| Incidence of diabetes in Asia | yes | none | none | yes | none | none | none | 3 | 1.63[1.09-2.43] | **⊕⊕⊝⊝** |

Table 9C: Reason for assessment of the quality of the body of evidence with GRADE

| Outcome | Risk of bias | Inconsistency | Imprecision | Indirectness | Publication bias | Magnitude of association | Residual confounding |
| --- | --- | --- | --- | --- | --- | --- | --- |
| Incidence of diabetes | The sample size of moderate-quality literature and low-quality literature is small. We consider the risk of bias acceptable. | Heterogeneity analyses showed moderate heterogeneity, with one paper (Obayashi, K,2020) deviating from the combined effects interval, and the inconsistency can be explained given the different types of studies combined and the low-quality rating of this study. | No study showed associations in the opposite direction to the same exposure outcome or confidence intervals that crossed clinical decision thresholds. | One article (Obayashi, K, 2020) included only the elderly, with population indirectness, and one article (Wang, C, 2023) determined the severity of exposure only by questionnaire, with exposure indirectness. | Funnel plot suggests no publication bias. | Escalation do not increase in reported effect sizes of 40%. | not report |
| Incidence of diabetes with low and moderate light pollution | Included literature was of high quality and judged to be at low risk of bias. | Heterogeneity analysis showed low heterogeneity. | No study showed associations in the opposite direction to the same exposure outcome or confidence intervals that crossed clinical decision thresholds. | one article (Wang, C, 2023) determined the severity of exposure only by questionnaire, with exposure indirectness. | Insufficient number of papers included. | Escalation do not increase in reported effect sizes of 40%. | not report |
| Incidence of diabetes with heavy light pollution | Included literature was of high quality and judged to be at low risk of bias. | Heterogeneity analysis showed low heterogeneity. | No study showed associations in the opposite direction to the same exposure outcome or confidence intervals that crossed clinical decision thresholds. | one article (Wang, C, 2023) determined the severity of exposure only by questionnaire, with exposure indirectness. | Insufficient number of papers included. | Escalation do not increase in reported effect sizes of 40%. | not report |
| Incidence of diabetes with Indoor light pollution | Although there was 1 low-quality paper (Obayashi, K,2014), which may have been biased, the sample was of such small proportion that the risk of bias was acceptable. | Heterogeneity analyses showed high heterogeneity, with one paper (Obayashi, K,2020) deviating from the combined effects interval, and the inconsistency can be explained given the different types of studies combined and the low-quality rating of this study. | No study showed associations in the opposite direction to the same exposure outcome or confidence intervals that crossed clinical decision thresholds. | One article (Obayashi, K, 2020) included only the elderly, with population indirectness, and one article (Wang, C, 2023) determined the severity of exposure only by questionnaire, with exposure indirectness. | Insufficient number of papers included. | Escalation do not increase in reported effect sizes of 40%. | not report |
| Incidence of diabetes with outdoor light pollution | Included literature was of high quality and judged to be at low risk of bias. | Heterogeneity analysis showed low heterogeneity. | No study showed associations in the opposite direction to the same exposure outcome or confidence intervals that crossed clinical decision thresholds. | All included literature PICO elements are straightforward. | Insufficient number of papers included. | Escalation do not increase in reported effect sizes of 40%. | not report |
| Incidence of diabetes in Europe and America | Included literature was of high quality and judged to be at low risk of bias. | Heterogeneity analyses showed moderate heterogeneity, with one study (Kim, M,2023) deviating from the combined effects interval, and the inconsistency can be explained given the different types of studies combined and the low-quality rating of this study. | No study showed associations in the opposite direction to the same exposure outcome or confidence intervals that crossed clinical decision thresholds. | one article (Wang, C, 2023) determined the severity of exposure only by questionnaire, with exposure indirectness. | Insufficient number of papers included. | Escalation do not increase in reported effect sizes of 40%. | not report |
| Incidence of diabetes in Asia | Inclusion of 1 medium-quality article and 1 low-quality article (Obayashi, K, 2014; Obayashi, K, 2020). | Heterogeneity analyses showed moderate heterogeneity, with one paper (Obayashi, K,2020) deviating from the combined effects interval, and the inconsistency can be explained given the different types of studies combined and the low-quality rating of this study. | No study showed associations in the opposite direction to the same exposure outcome or confidence intervals that crossed clinical decision thresholds. | One article (Obayashi, K, 2020) included only the elderly, with population indirectness. | Insufficient number of papers included. | Escalation do not increase in reported effect sizes of 40%. | not report |

Appendix Table 10: List of abbreviations

LAN: Light at Night

PROSPERO: Prospective Register of Systematic Reviews

CINAHL: Cumulative Index to Nursing and Allied Health Literature

PICOS: Patient, Intervention (Exposure), Comparison, Outcome, Study design

CI: confidence interval

NA: Not Applicable

NR: Not report

NOS: Newcastle-Ottawa scale

AHRQ: Agency for Healthcare Research and Quality scale

OR: Odds Ratio

RR: Risk Ratio

HR: Hazard Ratio

PR: Prevalence Ratio

I²: I-squared

GRADE: Grading System for Assessment, Development and Evaluation of Recommendations

Appendix figure 1: Light pollution and diabetes incidence subgroup forest plots

Figure 1A: area subgroup forest plot


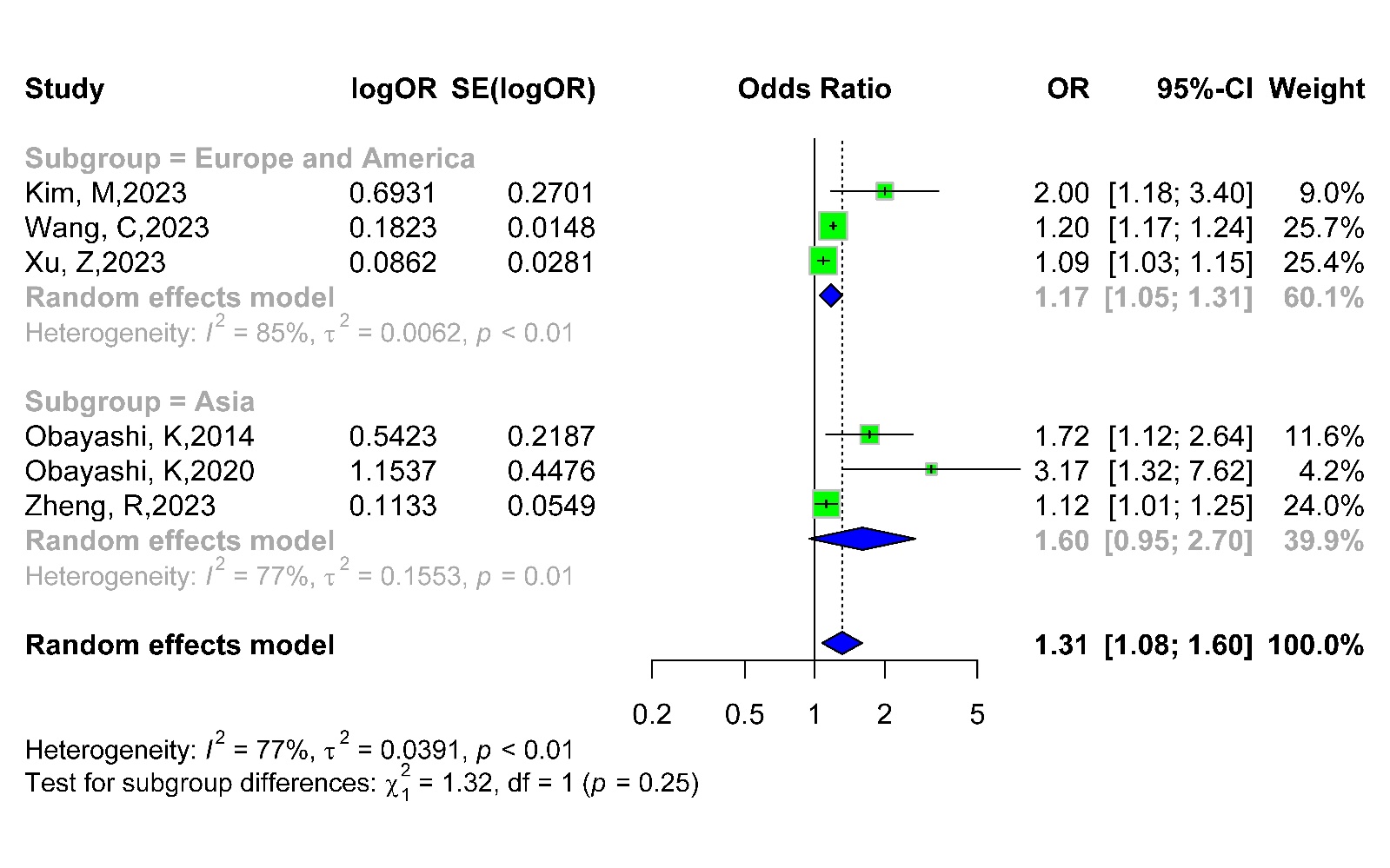


Figure1B: study type subgroup forest plot

**
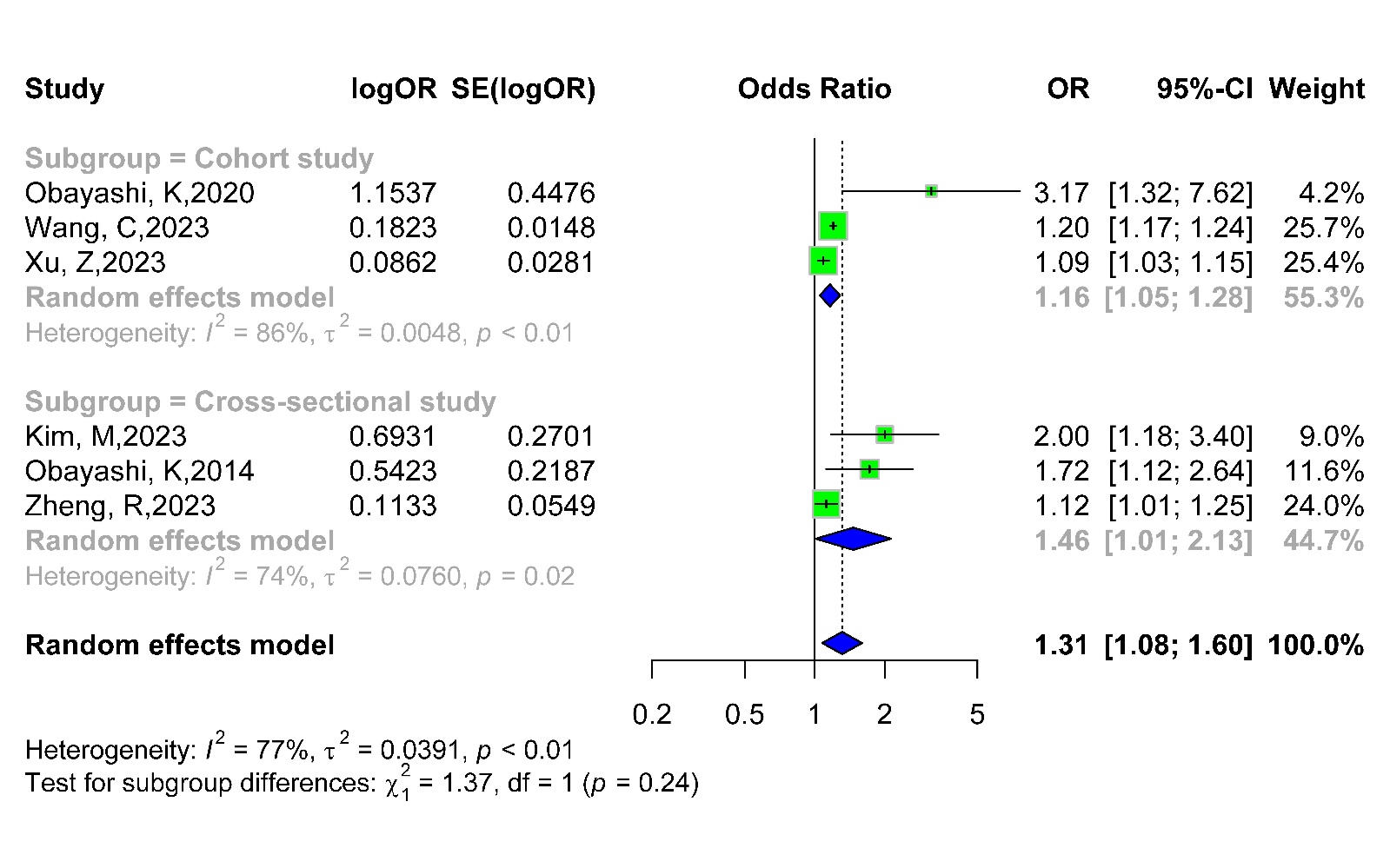
**

Figure1C: study quality subgroup forest plot


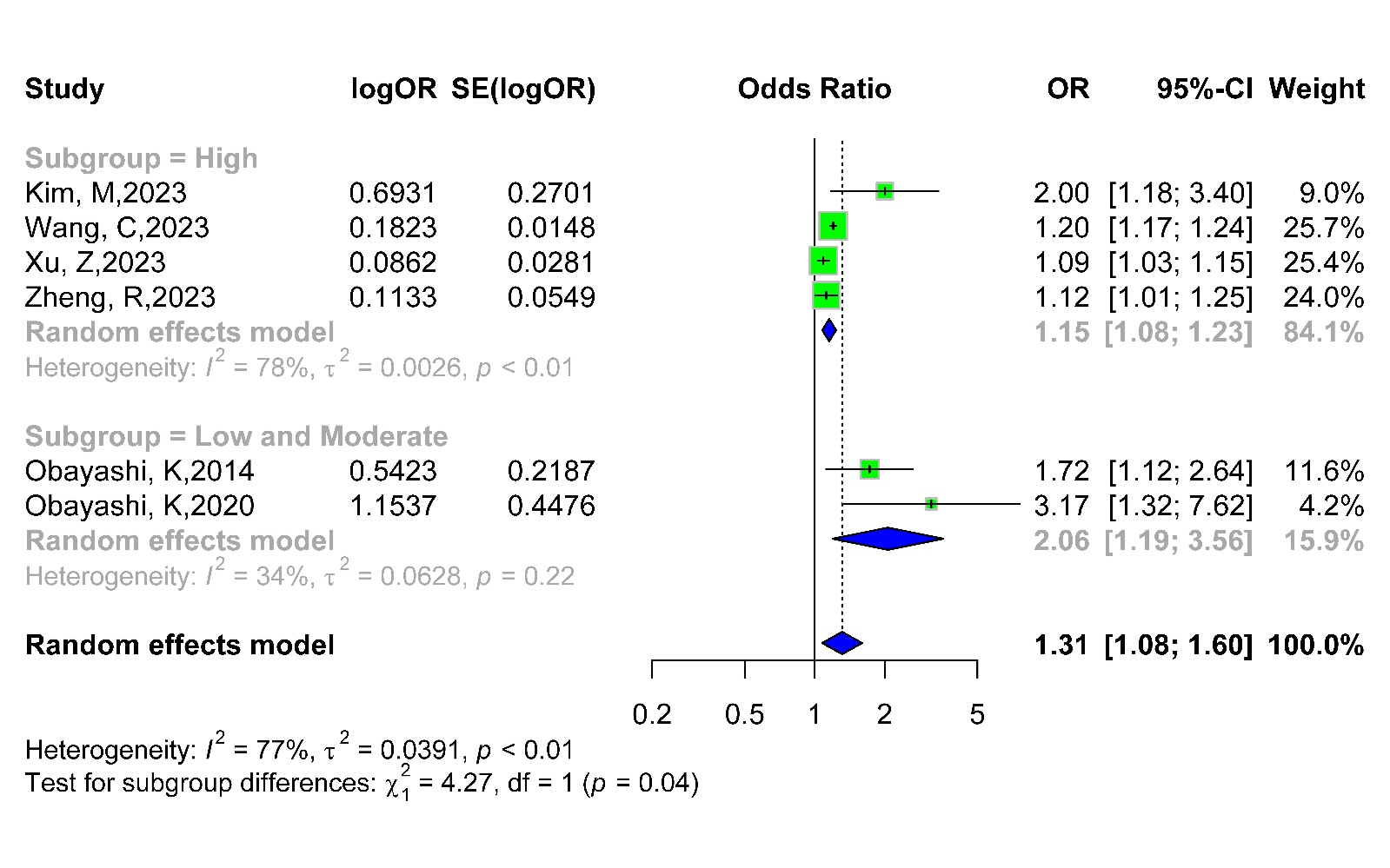


Figure1D: sample quantity subgroup forest plot

**
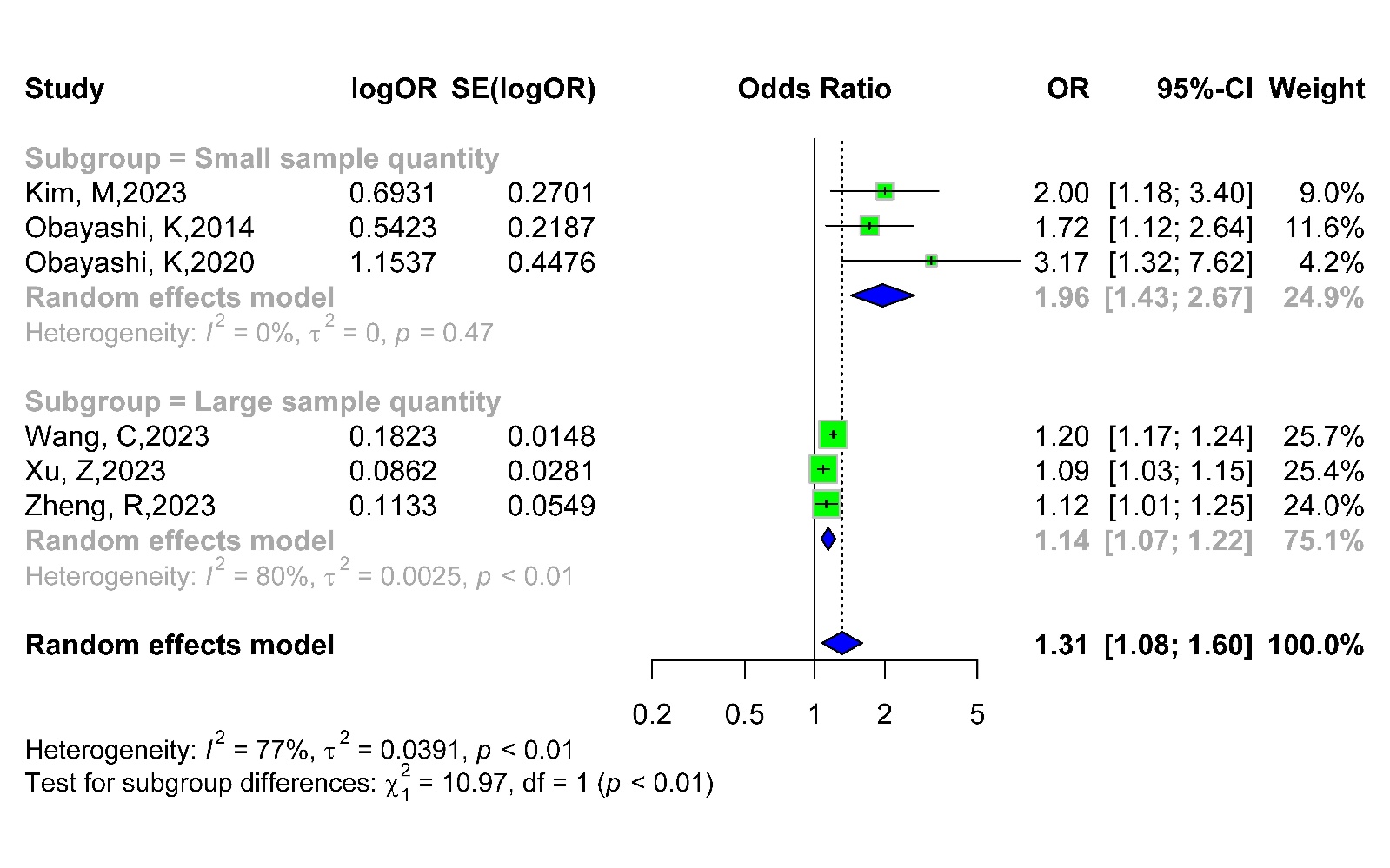
**

Appendix figure 2: Sensitivity analysis forest plot


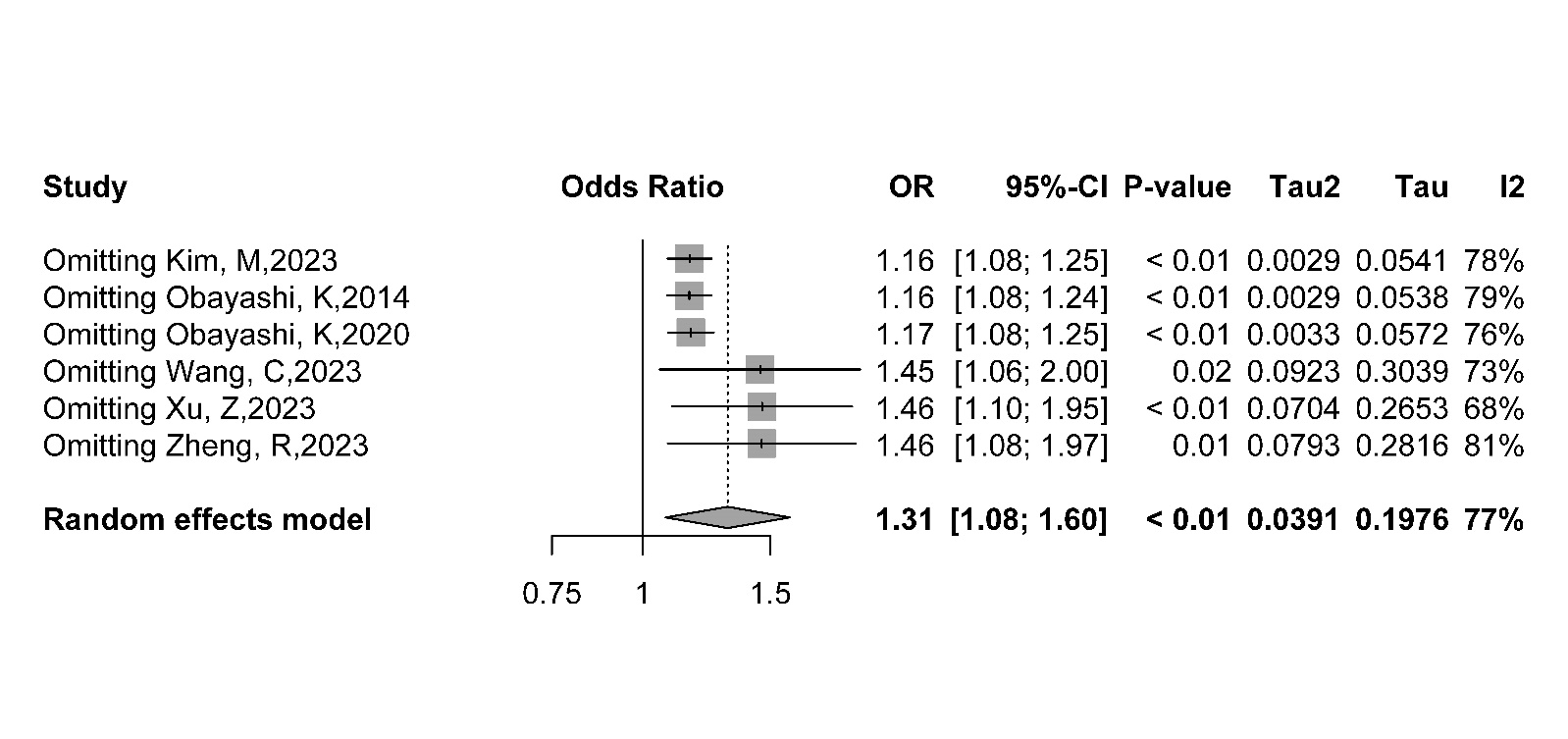

Supplement: Supplementary file 1 [file Data_Sheet_1.docx]
